# Linking species habitat and past palaeoclimatic events to evolution of the teleost innate immune system

**DOI:** 10.1098/rspb.2016.2810

**Published:** 2017-04-26

**Authors:** Monica Hongrø Solbakken, Kjetil Lysne Voje, Kjetill Sigurd Jakobsen, Sissel Jentoft

**Affiliations:** 1 Centre for Ecological and Evolutionary Synthesis (CEES), Department of Biosciences, University of Oslo, PO Box 1066, Blindern, 0316 Oslo, Norway; 2 Department of Natural Sciences, University of Agder, Kristiansand, Norway

**Keywords:** adaptive evolution, innate immunity, Toll-like receptors, gene loss, gene expansion, past climatic change

## Abstract

Host-intrinsic factors as well as environmental changes are known to be strong evolutionary drivers defining the genetic foundation of immunity. Using a novel set of teleost genomes and a time-calibrated phylogeny, we here investigate the family of Toll-like receptor (*TLR*) genes and address the underlying evolutionary processes shaping the diversity of the first-line defence. Our findings reveal remarkable flexibility within the evolutionary design of teleost innate immunity characterized by prominent *TLR* gene losses and expansions. In the order of Gadiformes, expansions correlate with the loss of major histocompatibility complex class II (*MHCII*) and diversifying selection analyses support that this has fostered new immunological innovations in *TLR*s within this lineage. In teleosts overall, *TLRs* expansions correlate with species latitudinal distributions and maximum depth. By contrast, lineage-specific gene losses overlap with well-described changes in palaeoclimate (global ocean anoxia) and past Atlantic Ocean geography. In conclusion, we suggest that the evolvability of the teleost immune system has most likely played a prominent role in the survival and successful radiation of this lineage.

## 1. Background

The evolutionary success of ray-finned fishes (class Actinopterygii) is characterized by large species radiations [1]. Actinopterygii comprises an exceptionally diverse group of fishes with species inhabiting numerous aquatic habitats spanning from Arctic to Antarctic oceans, deep-sea benthos to the shore, along coastlines and rivers as well as freshwater systems. Moreover, the high degree of diversity is mirrored in the array of life-history strategies, morphological varieties, distinct migratory behaviour and reproductive strategies displayed ([2,3] and references therein). The teleost lineage is the largest within the class of ray-finned fishes [4]. Genome sequencing efforts of non-model organisms have provided new insight into the extreme diversity of the teleost lineage including evidence for several alternate immunological strategies. The discoveries of the genetic loss of the major histocompatibility complex (*MHC*) class II pathway in Atlantic cod (*Gadus morhua*) as well as the functional loss in the more distant broadnosed pipefish (*Syngnathus typhle*) [5,6] show that *MHCII* is not crucial for the defence against pathogens and survival in some fish species. These findings are further supported in a recent study by Malmstrom *et al*. [7], which demonstrated that the loss of *MHCII* is shared by the entire Gadiformes lineage. Accompanying the loss of *MHCII,* highly variable *MHCI* copy number within the Gadiformes was reported, with several species having more than 40 copies including Atlantic cod found to have 80–100 copies [7,8]. Furthermore, it was hypothesized that the expanded repertoire of *MHCI* had undergone sub- or neofunctionalization as a possible adaptation to the *MHCII* loss. However, Malmstrom *et al*. also identified large numbers of *MHCI* in many Percomorphaceae lineages (all containing *MHCII*) demonstrating an extreme evolutionary plasticity of teleost immunity, and that it most likely is influenced by species habitat. Additional analyses revealed a correlation between high *MHCI* copy number and elevated speciation rates, and thus being a key to the success of this group of fishes [7].

The teleost immune system also displays important strategies with respect to the innate immune system such as the alternative set of Toll-like receptor (*TLR*) genes compared with other vertebrates [9–11]. Again, Atlantic cod is reported to be divergent compared with other investigated teleosts. In a recent study, the *TLR* repertoire in Atlantic cod was characterized and compared with that of other genome-sequenced fish species, revealing that Atlantic cod displays large gene expansions and several gene losses. These findings were attributed to the loss of *MHCII,* which may have boosted evolutionary innovation leading to a more complex *TLR* repertoire [12].

In general, it is the genetic basis of teleost alternative immunological strategies that has been investigated and studies beyond this tend to focus on genes related to the adaptive immune system. However, the underlying selective mechanisms driving the variety of immunological strategies observed and why they arose are poorly understood—especially for the innate immune system. Using genome assemblies from 66 teleost species, our aim was to characterize teleost *TLRs* with emphasis on the Gadiformes lineage and thereby investigate the possible link between the loss of *MHCII*, past and present environmental conditions and the genetic architecture of the innate immune system. We show that the teleost *TLR* repertoire contains an array of lineage-specific losses and expansions, with the Gadiformes lineage as an extreme outlier. Importantly, within the Gadiformes, we discovered expansions of *TLR* genes to be correlated with the loss of *MHCII* and to display different patterns of selection. Furthermore, in teleosts overall, we found that *TLR* copy number variation correlated with species latitudinal distribution in teleosts overall. By contrast, a weak correlation was found with species maximum depth for *TLR9* and *TLR22*. This suggests that there is a strong ongoing selection of the innate immune system linked to specific environmental and host-intrinsic factors. Furthermore, timing of the lineage-specific losses overlaps with well-described changes in palaeoclimate and continental drift, and hence unveils past adaptive signatures driving the genetic change within the teleost immune system. Our study reveals a remarkable evolutionary flexibility of teleost innate immunity, which has played an essential role in the survival and radiation of the teleost lineage.

## 2. Material and methods

### (a) Sequencing and assembly summary

**Figure 1.**
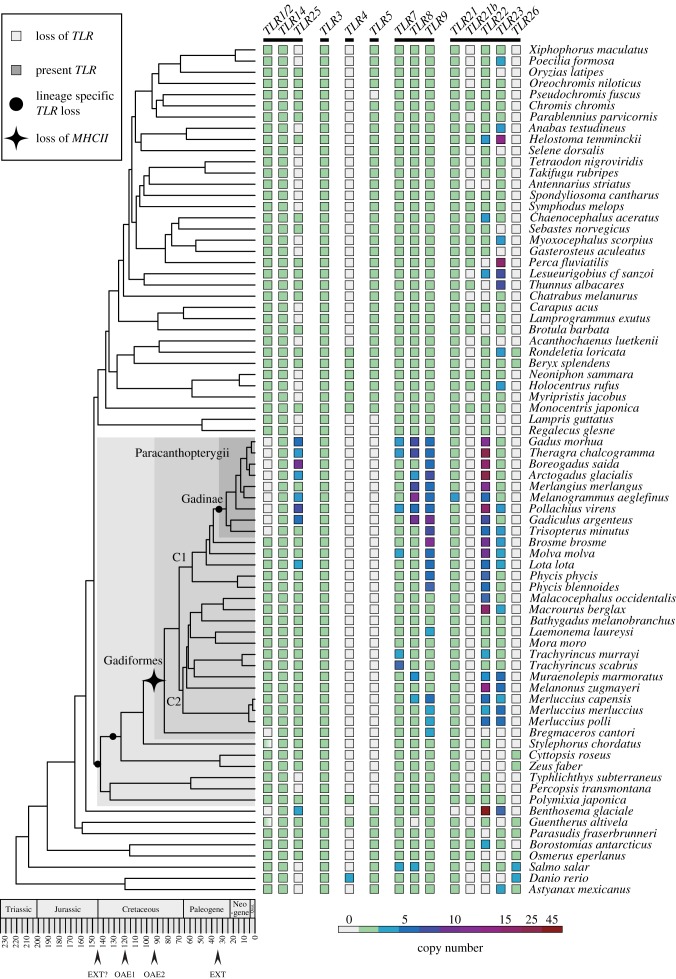
The *TLR* repertoires of 76 teleosts mapped onto a time-calibrated species phylogeny. All *TLRs* characterized in the new 66 teleost genomes as well as in 10 reference teleosts genomes (Ensembl and GenBank) mapped onto a species phylogeny generated by Malmstrom *et al*. The phylogeny demonstrates the loss of *MHCII* 110–64 Ma (branch range time, black star) reported by Malmstrom *et al*. Lineage-specific *TLR* losses are marked by black circles (Gadinae *TLR1/2*, Paracanthopterygii *TLR5* and *TLR21beta*). The individual species' repertoires are depicted with boxes where the coloration represents the number of copies of each individual *TLR*. The six major *TLR* families: *TLR1-family*, *TLR3-family*, *TLR4-family*, *TLR5-family*, *TLR7-family* and *TLR11-family* are indicated with black bars underneath the *TLR* names. See the electronic supplementary material, table S1 for copy number details. For *TLR1/2*, a gradient-filled box indicates the presences of either *TLR1* or *TLR2*. The Paracanthopterygiian lineage, Gadiformes order and Gadinae family display shaded grey backgrounds.

The 66 teleost genomes and species phylogeny were generated by Malmstrom *et al*. [7,13]. In short, DNA was isolated from 66 teleost species and subjected to Illumina HiSeq sequencing (2 × 150 bp paired-end reads) which after trimming resulted in an overall coverage between 9 and 34×. The genomes were assembled using the Celera Assembler. For the phylogenetic reconstruction, nine reference fish species were added from Ensembl together with *Salmo salar*. An alignment of 71 418 bp was used as input for phylogenetic reconstruction with the Bayesian software BEAST [14]. The phylogeny was made using BEAST combined with fossil time-calibration. Note: all timings derived from the phylogeny presented in this study include the confidence interval to illustrate the uncertainty underlying the time fossil calibration performed by Malmstrom *et al*. [7], and thus spans a longer time period than the branches depicted in the phylogeny (figure 1).

### (b) Gene searches

Protein query TIR (Toll/interleukin-1 receptor) domain sequences from Atlantic cod [12], all fish genomes available at Ensembl [15] and channel catfish [16–18], collectively representing all known vertebrate *TLR* genes to date, were used for TBLASTN searches towards the 66 fish genomes supplied by Malmstrom *et al.* (see below for parameters). *TLR* copy numbers for the Ensembl species were taken from [12]. The NCBI BLAST tool was used to search the *S. salar* genome (ICSASG_v2, GCA_000233375.4) with default settings using the same query sequences. TBLASTN from BLAST + 2.2.26 [19] was used with an *e*-value cut-off at 1*e*−10 (and in some cases lower, to capture the largest expansions), otherwise default settings. The number of detected TIR domains was counted for each *TLR* gene. Owing to the fragmented nature of the genomes, conservative estimates of copy numbers were used and are shown in the electronic supplementary material, table S1. These copy numbers form the foundation for the *TLR* repertoires depicted in figure 1.

Note on gene annotation: *TLR* gene annotation varies greatly between species. In this study, the following annotations are used (similar to that of [12]): *TLR1*, *TLR1/6* (in cases where annotation has not been provided and phylogeny cannot determine stronger homology towards *TLR1* or *TLR6*), *TLR2*, *TLR3*, *TLR4*, *TLR5*, *TLR6*, *TLR7*, *TLR8*, *TLR9*, *TLR10*, *TLR11*, *TLR12*, *TLR13*, *TLR14*, *TLR15*, *TLR16*, *TLR18* is by phylogeny determined to be *TLR14*, *TLR15*, *TLR16*, *TLR19* is by phylogeny determined to be *TLR26*, *TLR20* is by phylogeny determined to be *TLR26*, *TLR21*, *TLR22*, *TLR23*, *TLR25* and *TLR26*.

### (c) *TLR*, *MHC,* latitude and depth correlations using stochastic linear Ornstein–Uhlenbeck models for comparative hypotheses

For genes displaying more than four different gene copy numbers (*TLR8*, *TLR9*, *TLR22*, *TLR23*, *TLR25*), we ran SLOUCH—stochastic linear Ornstein–Uhlenbeck models for comparative hypotheses. This is a phylogenetic comparative method designed to study adaptive evolution of a trait along a phylogeny implemented in the R program SLOUCH [20–22]. The output of models analysed in SLOUCH can be summarized by a regression, which includes information on whether the analysed traits are evolving towards the estimated optima, how fast (or slow) this evolution is and how much of the trait variation that is explained by evolution towards these optima. We used SLOUCH to test whether *TLR* copy numbers have evolved towards optima that are influenced by the species' latitudinal distribution (values obtained from Fishbase.org [23]), species maximum depth (values obtained from Fishbase.org [23]) and evolutionary loss of the *MHCII* complex. We defined six latitudinal categories: 75, 50, 25, 0 (equator), −25 and −50. If a species' latitudinal distribution includes or crosses one of these categories, it was assigned to that respective category (multiple assignments are possible). Some species were not included in any of the categories owing to failure to cross the defined latitudes. Similarly, where data on depth were unavailable, species were excluded from the phylogeny resulting in a reduced tree used as input for SLOUCH.

The model of evolution in SLOUCH is based on an Ornstein–Uhlenbeck process and assumes that a trait (e.g. gene copy number) has a tendency to evolve towards a ‘primary’ optimum *Θ*. We assume that average copy number in a lineage can take any non-negative real number (i.e. intraspecies variation in copy numbers exist). A primary optimum is defined as the average optimal state that species will reach in a given environment when ancestral constraints have disappeared [20], at a rate proportional to a parameter *α*. As an example, in some of our analyses, we investigated whether species sharing the same latitudinal distribution have a tendency to evolve similar copy numbers for a given *TLR* locus. Lag in adaptation towards primary optima is quantified by a half-life parameter, *t*_1/2_ = ln(2)/*α*, which can be interpreted as the average time it takes a species to evolve half the distance from the ancestral (copy number) state towards the predicted optimal (copy number) state. For example, a half-life of zero signifies immediate adaptation of the trait to any change in the optimum for every lineage present in the phylogeny. A half-life above zero indicates adaptation is not immediate, with the amount of constrained evolution increasing with an increasing half-life. The model of evolution used in SLOUCH also includes a stochastic component with standard deviation *σ*, which can be interpreted as evolutionary changes in the trait (e.g. copy numbers) owing to unmeasured selective forces and genetic drift. This component of the model is reported as *v_y_* = *σ*^2^/2*α*, and can be interpreted as the expected residual variance when adaptation and stochastic changes have come to an equilibrium.

Our latitudinal categories, maximum depth and evolutionary losses of *MHCII* represent ‘niches’ and the model estimates one primary optimum for each niche included in any particular model. The different states of niches (e.g. presence and absence of *MCHII*) are known for all extant species in our phylogeny, but are unobserved for internal branches in the tree. We therefore mapped a separate state called *ancestral* to all internal nodes in the phylogeny to avoid having to infer uncertain primary optima. The method uses generalized least squares for estimation of the regression parameters (i.e. the influence of the predictor on the primary optimum) and maximum-likelihood for estimation of *α* and *σ*^2^ in an iterative procedure. For a full description of the model implemented in SLOUCH, see Hansen *et al*. [21]. All analyses were performed in R v. 3.0 [22].

We used SLOUCH to estimate the phylogenetic effect in the data. A phylogenetic effect indicates that some part of the variation in the trait is explained by shared ancestry (i.e. phylogeny), which means closely related species tend to have more similar trait values compared with more distantly related species. The phylogenetic effect can be estimated in SLOUCH by running a model without any predictor variables (i.e. no latitudinal categorical variables). The half-life parameter in such a model will represent an estimate for how important shared history is in explaining the distribution of trait means (average) on the phylogeny: a half-life of zero indicates that the trait data are not phylogenetically structured, while a half-life greater than 0 indicates that there exists an influence of phylogeny on the data. A phylogenetic effect can be owing to slowness of adaptation, adaptation towards phylogenetically structured optima or a combination of both. To investigate which of these scenarios we find support for, we contrasted the phylogenetic effect model with a model run with predictor variables (e.g. latitudinal distribution or maximum depth) using the bias-corrected Akaike information criterion (AICc), which balances goodness of fit (log-likelihood) with the number of parameters in the model (model complexity). The model with the lowest AICc value is the best supported. A better (lower) AICc value for a model including predictor variables indicate evidence for a scenario where the traits in our models are evolving towards optima that are shared by species across niches (e.g. the same latitudinal section). *r*^2^ was not used for assessing model support, but represents the amount of the total variation in the response trait (*TLR* gene copy number) that is explained by the optimal regression.

### (d) Diversifying selection analysis using mixed effects model of evolution and branch-site random effects likelihood

As there were different degrees of *TLR* gene expansions throughout our dataset, and because expansions were more prominent within the Gadiformes order, we wanted to determine if any individual positions within the coding sequence or certain lineages have been affected by diversifying selection. Owing to the fragmented nature of our dataset, this analysis was not feasible unless we selected a set of species as well as a set of *TLRs*. We selected nine species from the draft genome dataset: *Melanogrammus aeglefinus*, *Macrourus berglax* and *Muraenolepsis marmoratus* from the Gadiformes, *Stylephorus chordatus* which is a putative ancestral clade of Gadiformes, *Cyttopsis roseus* and *Zeus faber* from the Zeiformes (Gadiformes + *S. chordatus* sister clade), *Polymixia japonica* at the base of the Paracanthopterygii superorder, *Rondeletia loricata* and *Beryx splendens* as two closely related species outside the Paracanthopterygii. We also included *TLR* sequences from the second version of the Atlantic cod genome (GadMor2) as an additional Gadiformes representative [24]. Finally, we added the respective *TLR* sequences from fish species whose genomes are available through Ensembl [15]. Collectively, these species cover the entire range of the teleost phylogenetic tree obtained from Malmstrom *et al.* [7].

We selected three *TLR* genes for investigation: *TLR3*—a single copy gene present in all investigated teleosts, *TLR9*—expanded in most Gadiformes as well as present in all investigated teleosts and *TLR25*—mainly expanded in the C1 clade of the Gadiformes but also displaying both presence and absence patterns in our data. Collectively, these genes represent the range of different patterns observed. Query *TLR* sequences were identical to those used for the overall *TLR* characterization described above except the full-length protein sequence was used in a TBLASTN with an *e*-value cut-off equal to 1 × 10^−10^ and otherwise default parameters towards the draft genomes and GadMor2. The target unitigs (draft genomes) and linkage group (GadMor2) regions were extracted and aligned towards the coding sequences obtained from Ensembl using ClustalW in MEGA5 [25]. The resulting alignment was manually curated to ensure that the reading frame was maintained. We chose to only investigate the ecto-domain of the TLR as the transmembrane and TIR domain are known to be under purifying selection. For all alignments, the coverage of unitig sequence was variable. Therefore, the alignments were divided into sections to obtain alignments with the least amount of missing data. This resulted in one alignment for *TLR3*, two for *TLR9* and four for *TLR25*. The alignments are available in our GitHub repository.

These alignments were uploaded to www.datamonkey.org [26,27] where we performed model selection analysis to find the best fitting model of nucleotide evolution for each of the alignments (reported in the electronic supplementary material). We then performed Mixed Effects Model of Evolution (MEME) analysis on all alignments as well as Branch-Site Random Effects Likelihood (BSR) analysis on *TLR9* and *TLR25* alignments allowing for the generation of gene trees based on the alignments. MEME is based on the ratio between non-synonymous to synonymous substitutions where this ratio can vary from site to site as well between lineages. In this way, MEME can detect both pervasive and episodic positive (diversifying) selection. MEME compares its estimates with a null hypothesis for which all sites are evolving neutrally (worst-case scenario) and thus, the results given by MEME are conservative estimates. BSR is also based on the ratio between non-synonymous to synonymous substitutions. MEME implements this analysis for each individual site, but we also ran BSR alone to obtain on overall impression of any likely diversifying selection affecting lineages or individual branches. By contrast, in BSR, there is no need to define any branches *a priori* as neutral or under negative selection. Thus, detecting episodic diversifying selection in a few sites or in a few lineages becomes more reliable by using BSR [28,29].

## 3. Results

Mapping all the identified teleost *TLRs*—extracted from the 66 genome assemblies—onto the phylogeny of Malmstrom *et al.* [7] demonstrates the presence of comprehensive *TLR* repertoires in all investigated teleosts (figure 1) similar to that found in other vertebrates [9,11,12]. However, most notable was the observation of three lineage-specific gene losses, several lineage-specific gene expansions and a substantial number of recorded species-specific repertoire variants (figure 1). Specifically, *TLR1/2* are lost from the Gadinae (40–16 Ma) in addition to being completely or partially lost in *Bregmaceros cantori*, *Benthosema glaciale*, *S. chordatus and Guentherus altivela*. *TLR5* is lost from the entire Paracanthopterygii superorder and the order Lampridiformes (175–130 Ma) in addition to *Pseudochromis fuscus*. Further, we discovered a new *TLR*, here annotated as *TLR21beta* based on sequence homology, which is also absent in all Paracanthopterygiian species with the exception of *P. japonica,* and Lampridiformes. However, in contrast with *TLR5*, the presence of *TLR21beta* does not follow any clear phylogenetic pattern outside Paracanthopterygii/Lampridiformes (figure 1). The Gadinae is the only clade consistent with the recently reported alternative *TLR* repertoire in Atlantic cod [5,12] owing to the prominent gene losses of *TLR1/2*.

Three *TLRs* are found in all species; *TLR3*, *TLR14* and *TLR21*, the latter with the exception of *Be. glaciale*. Within the Gadiformes, we find gene expansions for *TLR7*, *TLR8*, *TLR9*, *TLR22*, *TLR23* and *TLR25*, especially within the C1 clade (the Gadiformes segregate into two distinct clades here named C1 and C2 (figure 1)). Outside the Gadiformes, *TLR25* displays no obvious phylogenetic pattern. This is in contrast with *TLR7*, *TLR8* and *TLR9* which are present in all species with the exception of a single *TLR8* loss in *G. altivela*. *TLR22* and *TLR23* are found in all Gadiformes except in *Br. cantori* and show a substantial degree of gene expansion within the Gadiformes lineage—particularly for *TLR22*. Outside the Gadiformes, the expansion of *TLR22* is less pronounced, whereas, by contrast, *TLR23* is frequently expanded. However, *TLR22* and *TLR23* display phylogenetically non-structured patterns of presence and gene loss outside the Gadiformes order (figure 1; electronic supplementary material, table S1). Finally, there are two rare teleost *TLRs*, i.e. *TLR4* and *TLR26*. *TLR4* is found in the Holocentriformes and in three out of four Beryciformes species in addition to *Danio rerio*, *P. japonica* and *G. altivela*. *TLR26* is mainly found in species basal to the Gadiformes and in two Beryciformes: *R. loricata* and *B. splendens* (figure 1; electronic supplementary material, table S1).

**Figure 2.**
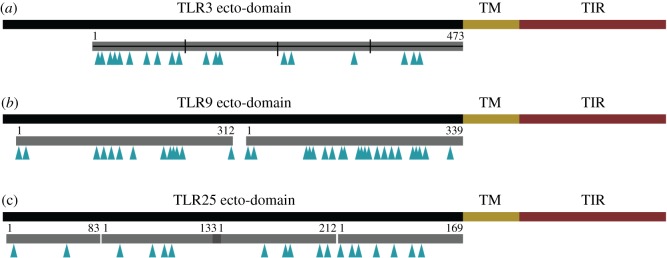
Overview of sites reported by the MEME analysis performed on *TLR3*, *TLR9* and *TLR25* in the selected species. A schematic drawing of the *TLR3* (*a*), *TLR9* (*b*) and *TLR25* (*c*) protein domains with the ecto-domain (dimerization and ligand interaction), transmembrane (TM) domain and TIR domain (signalling domain). Only the ecto-domain was subjected to selection analysis as the TM and TIR domains are known to be under purifying selection. Grey boxes indicate which parts of the ecto-domain were included in the alignment and also show how many alignments were generated per gene. There is a 22 codon overlap between TLR25 section 2 and 3. Arrows indicate sites reported by the MEME analysis. For site details, see the electronic supplementary material. (Online version in colour.)

To identify episodic diversifying selection, MEME and BSR selection analyses were performed on the ecto-domain of three *TLR* representatives—*TLR3*, *TLR9* and *TLR25*. MEME reported 19 sites for *TLR3*, 35 sites for *TLR9* and 18 sites for *TLR25* likely to have experienced diversifying selection (figure 2). The BSR analysis identified multiple nodes and branches encompassing most *TLR9* paralogues in the Gadiformes (mainly Gadinae) subject to diversifying selection. Diversifying selection was also detected in one of the *TLR25* alignments at one node and on one branch encompassing some of the Gadinae *TLR25* paralogues (electronic supplementary material, figures S1 and S2).

Associations between specific *TLR* expansions, species latitudinal distributions, species maximum depth as well as the absence of *MHCII*—specific for the Gadiformes lineage (figure 1)—were further investigated using SLOUCH [21]. Models using the specified latitudinal categories as predictor variables showed that latitude explained 19–32% of the *TLR* copy number variation for *TLR8*, *TLR9*, *TLR22* and *TLR25* (table 1), whereas species maximum depth explained 4–10% of the variation seen in *TLR9* and *TLR22* (electronic supplementary material). Especially northern latitudinal categories were found to be associated with higher copy numbers of *TLR8*, *TLR22* and *TLR25*, while increased copy numbers of *TLR9* were associated with more tropical latitudes—particularly in the equatorial region (table 1; electronic supplementary material, table S1). However, for *TLR23*, there was no indication that the copy number has evolved as a consequence of changes in latitude or depth (table 1, data not shown for depth correlation). Moreover, within the Gadiformes lineage, we found strong support for scenarios where *TLR8*, *TLR9*, *TLR22* and *TLR25* have evolved additional gene copies with the loss of *MHCII* explaining between 14% and 27% of the copy number variation (table 2). The explained variation in copy numbers was 3–6% larger (compared with latitude alone) and 3–16% larger (compared with *MHCII* loss alone) when we ran models where copy numbers of *TLR8*, *TLR22* and *TLR25* evolved towards optima jointly defined by latitudinal categories and presence/absence of *MHCII*. This indicates that both latitude and loss of *MHCII* have contributed to the expansion of these *TLRs.* However, we were not able to distinguish the relative contribution of *MHCII* and latitude, respectively. This is contrary to the striking result obtained for *TLR9* where the combination of latitude and loss of *MHCII* explained 50% of the copy number variation—compared with 20% and 22% for latitude and *MHCII* loss separately (table 2).

**Table 1.** Phylogenetic comparative analyses of the evolution of *TLR* copy numbers in relation to species latitudinal distributions using SLOUCH. (For each model, we show the phylogenetically corrected *r*^2^, and the AICc score. AICc balances goodness of fit (log-likelihood) with the number of parameters in the model (model complexity). The model with the lowest AICc value is the best supported. *r*^2^ represents the amount of the total variation that is explained by the model. Detailed output from each model is given in the electronic supplementary material. The model called ‘phylogeny’ does not include any explanatory variables and is given as a reference point for comparison to models with predictor variables. The best AICc scores and corresponding *r*^2^ values for each of the investigated TLR expansion are italicized.)

category	*TLR8*		*TLR9*		*TLR22*		*TLR23*		*TLR25*	
	AICc	*r*^2^	AICc	*r*^2^	AICc	*r*^2^	AICc	*r*^2^	AICc	*r*^2^
phylogeny	266.41	0.00	243.91	0.00	430.27	0.00	307.65	0.00	241.36	0.00
group 75 latitude	260.29	18.32	239.07	18.91	*418.86*	*24.63*	311.72	0.96	*226.61*	*32.26*
group 50 latitude	*259.75*	*19.02*	240.67	15.49	427.26	13.70	310.88	2.30	232.46	21.96
group 25 latitude	259.98	18.72	240.34	17.24	429.86	8.86	*307.22*	*7.91*	233.31	20.89
group 0 latitude	259.90	20.13	*238.24*	*19.99*	427.05	13.99	311.27	1.69	232.56	21.84
group −25 latitude	260.06	18.63	239.78	16.69	429.34	9.62	309.78	4.00	233.38	20.80
group −50 latitude	260.31	16.35	240.16	16.18	429.62	9.21	311.54	1.24	233.45	20.71

**Table 2.** Phylogenetic comparative analyses of the evolution of *TLR* copy numbers in relation to species latitudinal distributions and MHCII status using SLOUCH. (For each model, we show the phylogenetically corrected *r*^2^, and the AICc score. AICc balances goodness of fit (log-likelihood) with the number of parameters in the model (model complexity). The model with the lowest AICc value is the best supported. *r*^2^ represents the amount of the total variation that is explained by the model. Detailed output from each model is given in the electronic supplementary material. The model called ‘phylogeny’ in table 1 does not include any explanatory variables and is given as a reference point for comparison to models with predictor variables. The best AICc scores and corresponding *r*^2^ values for each of the investigated TLR expansion are italicized.)

category	*TLR8*		*TLR9*		*TLR22*		*TLR23*		*TLR25*	
	AICc	*r*^2^	AICc	*r*^2^	AICc	*r*^2^	AICc	*r*^2^	AICc	*r*^2^
group MHCII	259.43	19.44	239.01	22.41	427.99	14.53	328.13	2.65	231.30	26.94
group MHCII + group 75 lat.	264.37	19.52	240.39	31.`32	*420.41*	*30.23*	315.34	3.21	*228.98*	*35.08*
group MHCII + group 50 lat.	*262.31*	*22.16*	243.11	25.72	431.07	17.15	314.69	4.23	235.60	27.76
group MHCII + group 25 lat.	263.76	20.32	239.13	32.69	431.69	16.32	*311.21*	*9.41*	234.82	28.67
group MHCII + group 0 lat.	*261.00*	*27.11*	*228.48*	*53.53*	430.16	18.36	314.90	3.92	233.63	30.02
group MHCII + group −25 lat.	263.37	20.82	230.06	52.33	432.07	15.80	313.49	6.01	234.84	28.64
group MHCII + group −50 lat.	264.39	19.50	240.15	29.17	432.19	15.63	314.92	3.81	235.60	27.76

## 4. Discussion

Overall, vertebrate and teleost genome duplications may explain some of the teleost *TLR* repertoire variation demonstrated here with respect to gene expansions. However, the extreme numbers seen for some of the *TLR* expansions within the Gadiformes indicate that these genes have undergone additional lineage-specific duplication events—a phenomenon also seen for other genes in teleost species [30]. Gene duplicates preserved after a duplication event commonly undergo neo- or subfunctionalization ([31] and references therein). In Atlantic cod, we have previously demonstrated that the *TLR* expansions and their paralogues show signs of diversifying selection. For some expansions, this was indicative of neofunctionalization owing to high numbers of sites under selection in putative dimerization and ligand-interacting regions. For other expansions, it was more indicative of subfunctionalization owing to fewer sites under selection combined with tissue-specific expression patterns [12]. The selection analyses on the chosen *TLR* representatives demonstrated that *TLR3* and *TLR25* display similar amounts of sites subject to diversifying selection, despite their highly different patterns in our dataset (single copy present in all species versus expanded in Gadiformes combined with both presence and absence in the remaining species). By contrast, *TLR9* displayed almost double the number of sites reported as under diversifying selection (figure 2). In the human system, and by proxy in teleosts, the TLR3 protein is located to the endosomal membranes and signals for an antiviral response upon interaction with double-stranded RNA [32]. It has recently been demonstrated that mammalian TLR3 also can detect structured RNAs [33]. This could explain the presence of sites under diversifying selection in fish *TLR3* adapting the protein towards different structured RNAs or other possible ligands not presently known.

*TLR25* is a relatively newly identified fish-specific *TLR* where ligand and subcellular localization is yet to be determined [17]. We have earlier suggested that this TLR in Atlantic cod is located to the cell surface and interacts with ligands similar to other TLR1-family members (*TLR1*, *TLR2* and *TLR6*)—such as bacterial or parasitic lipoproteins [12]. In humans, TLR1, TLR2 and TLR6 form both homo- and heterodimers actively increasing their ligand repertoire [32]. Gadinae do not have *TLR1*, *TLR2* or *TLR6* (figure 1) and thus, in their case, *TLR14* and the expanded *TLR25* may be a replacing set of TLR1-family members. In the remaining Gadiformes, both *TLR1*, *TLR2* as well as *TLR14* and *TLR25* are present (figure 1). The MEME analysis reported a similar number of sites under diversifying selection in *TLR25* compared with *TLR3,* which could suggest that they are subjected to similar selective pressures. However, the BSR analysis indicated that nodes and branches representing only some of the Gadinae *TLR25* paralogues are subject to diversifying selection (electronic supplementary material, figure S2). Overall, this demonstrates that *TLR25* paralogues may be affected by different selection pressures within expansions, whereas *TLR25* generally is adapted towards unknown species-specific factors similar to that of *TLR3*.

In Atlantic cod, *TLR9* paralogues showed clear signs of diversifying selection and differences in expression patterns [12]. The MEME analysis reported a large amount of sites under diversifying selection and the BSR analysis strongly indicates diversifying selection on nodes and branches leading to different clades of Gadinae *TLR9* paralogues. In humans, TLR9 interacts with unmethylated single-stranded CpG DNA, both viral and bacterial, within the endosomal track in a highly sequence-dependent manner. However, dependent on the sequences, TLR9-ligand interaction can result in both antagonistic and agonistic signalling [34] suggesting a regulatory role. Diversification of *TLR9* paralogues indicates adaptation towards lineage-specific pathogen loads or diversity within Gadiformes. It may also suggest a larger regulatory role for *TLR9* in this lineage. Furthermore, in mammals, TLR9 signalling can induce MHCI antigen cross-presentation [35] which overlaps with the hypothesized subfunctionalization of some *MHCI* copies in Gadiformes [7]. Overall, our findings demonstrate that *TLR9* paralogues have experienced a different selection pressure compared with *TLR25* paralogues. Collectively, the gene expansions observed in Gadiformes, as well as in teleosts overall, are probably subject to different levels of neo- and subfunctionalization contributing to the further adaptation of the teleost innate immune system. Extreme northern or southern distributions are proxy indicators for temperature as these regions are cooler but also have undergone a larger degree of palaeoclimatic changes compared with the more tropical regions [36]. The observed expansions for *TLR7, TLR8*, *TLR9*, *TLR22, TLR23* and *TLR25*, especially within the Gadiformes, indicate selection towards higher copy number optima. This could potentially be explained by different pathogen loads or pathogen community compositions connected to highly variable palaeoclimatic arctic environments. We found correlations between increased copy number of *TLR8*, *TLR22* and *TLR25* with more northern species distributions (table 1). By contrast, *TLR9* showed higher optimal copy numbers in tropical regions—especially combined with the loss of *MHCII* (tables 1 and 2), most probably driven by the specific biotic or abiotic factors encountered in the tropics. Collectively, our findings indicate that, for the Gadiformes, both the palaeogeographical distribution (reflecting the environments these species have inhabited through time) and the loss of *MHCII* have been vital drivers for the expansion of *TLR8*, *TLR22*, *TLR25* and in particular *TLR9*.

By using a dated phylogeny, we find that the successive alterations to the teleost immune system occurred in periods with substantial palaeoclimatic fluctuations as well as oceanographic changes owing to continental drift. Such events are often associated with periods of extinction followed by population diversification and subsequent speciation enabling the invasion of new niches [37,38]. Our data suggest that the overall loss of *TLR5* (previously reported [39]) and *TLR21beta* (175–130 Ma) overlap the Jurassic–Cretaceous (J–K) boundary (figure 1). Although this transition between geological periods does not harbour any well-defined events, there is accumulating evidence of both species extinctions and radiations [40–44]. The loss of *TLR5* and *TLR21beta* may have occurred as adaptations to new habitats such as the expanding Central Atlantic Ocean. Even though both *TLR5* and *TLR21b* display lineage-specific loss, their presence/absence pattern outside the Paracanthopterygii (figure 1) indicate that they have experienced different selection pressures before the J–K boundary.

Within the Gadiformes clade, we find that the loss of *MHCII* coincides with the overall gene expansion patterns of *TLR7*, *TLR8*, *TLR9*, *TLR22*, *TLR23* and *TLR25*, spanning a total interval 110–64 Ma. This further overlaps with the early–late Cretaceous transition which includes one of the late Cretaceous global anoxia events (95 Ma). This anoxic environment, although probably allowing a small degree of specialized adaptation, generally deprived the deep seas of species [45,46]. Anoxic conditions led to higher extinction rates during this time period [47–50], fitting with the metabolic cost scenario proposed to promote the loss of *MHCII* [51]. In this scenario, the benefits of maintaining the MHCII system in some environments could not compensate for the metabolic cost of expressing it. Coinciding with the anoxic event is the further northward opening of the Central Atlantic Ocean [52] and the propagation of the South Atlantic Ocean to meet the Central Atlantic Ocean [53–55]. The stress imposed by global ocean anoxia therefore appears simultaneously with the appearance of new habitats. Further, this time period is associated with a decrease in bony fish family richness, indirectly derived from fossil data [56], indicating that these secondary changes to the Gadiformes immune system may have had slightly more adverse effects here compared with the initial ones occurring at the J–K boundary. However, this probably had a positive effect supporting species survival and radiation in the long term. The more recent loss of *TLR1/2* from the Gadinae subfamily (40–16 Ma) is probably a temperature-driven adaptation caused by an abrupt cooling of global climate and loss of habitat owing to the drastic decrease in eustatic sea levels approximately 34 Ma [50,57,58] overlapping with the opening of the North Atlantic Ocean between Greenland and Norway [52].

## 5. Conclusion

Overall, our findings reveal unprecedented variability within the teleost innate immune system, particularly within the Gadiformes, characterized by significant gene expansions and losses. Intriguingly, we find that higher copy numbers of *TLRs* correlate with species latitudinal distribution and the loss of *MHCII*. Further evidence of diversifying selection indicates that the paralogues probably experience different selection pressures. The successive nature of these changes to the ancestral teleost immune system, combined with the extensive evolvability of the innate immune system described here, have probably contributed to the overall survival and successful radiation of this lineage.

## Supplementary Material

Supplementary information

## Data accessibility

All novel teleost sequence and genome resources are available at European Nucleotide Archive (ENA) and the Dryad digital repository, submitted by the Malmstrom *et al.* [7]: all raw data (sequencing reads) are available at ENA with study accession number PRJEB12469 (sample identifiers ERS1199874-ERS1199939). Genome assemblies, available at Dryad, exist in two versions (UTGs and scaffolds) under http://dx.doi.org/10.5061/dryad.326r8. All additional resources needed to generate the findings presented here are available in our GitHub repository including, but not limited to, scripts, BLAST and SLOUCH output files: https://github.com/MonicaSolbakken/TLR [7,13].
