# Supplementary material for: Linking species habitat and past palaeoclimatic events to evolution of the teleost innate immune system
Source: Proc Biol Sci. 2017 Apr 26;284(1853):20162810. doi: 10.1098/rspb.2016.2810 (PMC5413918; doi:10.1098/rspb.2016.2810)
Supplement: Supplementary information [file rspb20162810supp1.pdf]

Supplementary information for manuscript:

Linking species habitat and past paleoclimatic events to evolution of the teleost innate immune system

Monica Hongrø Solbakken[1*], Kjetil Lysne Voje[1], Kjetill Sigurd Jakobsen[1], Sissel Jentoft[1,2]*

Journal: Proceedings of the Royal Society B

Main manuscript: 10.1098/rspb.2016.2810

Contents

Linking species habitat and past paleoclimatic events to evolution of the teleost innate immune system 1

Supplementary table 1 Overview of *TLR* copy number and northern and southern latitude boundaries collected from Fishbase. 4

Supplementary table 2 All query TIR domain sequences used for teleost *TLR* characterization derived from Ensembl.org v83. and GenBank. 10

SLOUCH results 21

BEST ESTIMATES & MODEL FIT – TLR8 phylogeny 21

BEST ESTIMATES & MODEL FIT – TLR8 group 0 22

BEST ESTIMATES & MODEL FIT – TLR8 group 25 23

BEST ESTIMATES & MODEL FIT – TLR8 group 50 24

BEST ESTIMATES & MODEL FIT – TLR8 group 75 25

BEST ESTIMATES & MODEL FIT – TLR8 group minus 25 26

BEST ESTIMATES & MODEL FIT – TLR8 group minus 50 27

BEST ESTIMATES & MODEL FIT – TLR8 MHCII 28

BEST ESTIMATES & MODEL FIT – TLR8 group 0 + MHCII 29

BEST ESTIMATES & MODEL FIT – TLR8 group 25 + MHCII 30

BEST ESTIMATES & MODEL FIT – TLR8 group 50 + MHCII 31

BEST ESTIMATES & MODEL FIT – TLR8 group 75 + MHCII 32

BEST ESTIMATES & MODEL FIT - TLR8 group minus 50 + MHCII 33

BEST ESTIMATES & MODEL FIT – TLR8 group minus 25 + MHCII 34

BEST ESTIMATES & MODEL FIT – TLR9 phylogeny 35

BEST ESTIMATES & MODEL FIT – TLR9 group 0 36

BEST ESTIMATES & MODEL FIT – TLR9 group 25 37
BEST ESTIMATES & MODEL FIT – TLR9 group 50 38
BEST ESTIMATES & MODEL FIT – TLR9 group 75 39
BEST ESTIMATES & MODEL FIT – TLR9 group minus 25 40
BEST ESTIMATES & MODEL FIT – TLR9 group minus 50 41
BEST ESTIMATES & MODEL FIT – TLR9 + MHC 42
BEST ESTIMATES & MODEL FIT – TLR9 group 0 + MHCII 43
BEST ESTIMATES & MODEL FIT – TLR9 group 25 + MHCII 44
BEST ESTIMATES & MODEL FIT – TLR9 group 50 + MHCII 45
BEST ESTIMATES & MODEL FIT – TLR9 group 75 + MHCII 46
BEST ESTIMATES & MODEL FIT – TLR9 group minus 25 + MHCII 47
BEST ESTIMATES & MODEL FIT – TLR9 group minus 50 + MHCII 48
BEST ESTIMATES & MODEL FIT – TLR22 phylogeny 49
BEST ESTIMATES & MODEL FIT – TLR22 group 0 50
BEST ESTIMATES & MODEL FIT – TLR22 group 25 51
BEST ESTIMATES & MODEL FIT – TLR22 group 50 52
BEST ESTIMATES & MODEL FIT – TLR22 group 75 53
BEST ESTIMATES & MODEL FIT – TLR22 group minus 25 54
BEST ESTIMATES & MODEL FIT – TLR22 group minus 50 55
BEST ESTIMATES & MODEL FIT – TLR22 + MHCII 56
BEST ESTIMATES & MODEL FIT – TLR22 group 0 + MHCII 57
BEST ESTIMATES & MODEL FIT – TLR22 group 25 + MHCII 58
BEST ESTIMATES & MODEL FIT – TLR22 group 50 + MHCII 59
BEST ESTIMATES & MODEL FIT – TLR22 group 75 + MHCII 60
BEST ESTIMATES & MODEL FIT – TLR22 group minus 25 + MHCII 61
BEST ESTIMATES & MODEL FIT – TLR22 group minus 50 + MHCII 62
BEST ESTIMATES & MODEL FIT – TLR25 phylogeny 63
BEST ESTIMATES & MODEL FIT – TLR25 group 0 64
BEST ESTIMATES & MODEL FIT – TLR25 group 25 65
BEST ESTIMATES & MODEL FIT – TLR25 group 50 66
BEST ESTIMATES & MODEL FIT – TLR25 group 75 67
BEST ESTIMATES & MODEL FIT – TLR25 group minus 25 68
BEST ESTIMATES & MODEL FIT – TLR25 group minus 50 69
BEST ESTIMATES & MODEL FIT – TLR25 + MHCII 70

BEST ESTIMATES & MODEL FIT – TLR25 group 0 + MHCII 71
BEST ESTIMATES & MODEL FIT – TLR25 group 25 + MHCII 72
BEST ESTIMATES & MODEL FIT – TLR25 group 50 + MHCII 73
BEST ESTIMATES & MODEL FIT – TLR25 group 75 + MHCII 74
BEST ESTIMATES & MODEL FIT – TLR25 group minus 25 + MHCII 75
BEST ESTIMATES & MODEL FIT – TLR25 group minus 50 + MHCII 76
BEST ESTIMATES & MODEL FIT – TLR9 phylogenetic effect 77
BEST ESTIMATES & MODEL FIT – TLR9 maximum depth 78
BEST ESTIMATES & MODEL FIT – TLR22 phylogenetic effect 80
BEST ESTIMATES & MODEL FIT – TLR22 maximum depth 81
Supplementary table 3 Diversifying selection analyses - results from MEME (mixed effects model of evolution) 83
Supplementary figure 1 86
Supplementary figure 2 87

Supplementary table 1 Overview of *TLR* copy number and northern and southern latitude boundaries collected from Fishbase.

Latin Name	TLR 1/6	TLR 2	TLR 3	TLR 4	TLR 5	TLR 7	TLR 8	TLR 9	TLR 14	TLR 21	TLR 21_beta	TLR 22	TLR 23	TLR 25	TLR 26	North.	South.	Average
Arctogadus glacilis	0	0	1	0	0	1	3	7	1	2	0	22	1	4	0	87	69	78
Boreogadus saida	0	0	1	0	0	1	1	6	1	1	0	16	1	10	0	87	52	69.5
Trisopterus minutus	0	0	1	0	0	2	1	8	1	2	0	6	3	1	0	66	28	47
Pollachius virens	0	0	1	0	0	3	5	5	1	1	0	15	3	7	0	77	33	55
Melanogrammus aeglefinus	0	0	1	0	0	1	12	5	1	3	0	6	2	4	0	79	35	57
Merlangius merlangus	0	0	1	0	0	2	7	6	1	1	0	11	1	2	0	72	35	53.5
Theragra chalcogramma	0	0	1	0	0	3	7	5	1	1	0	27	1	4	0	68	34	51
Gadiculus argentus	0	0	1	0	0	1	9	10	1	2	0	7	2	5	0	74	24	49
Phycis phycis	1	1	1	0	0	1	1	5	1	2	0	8	2	1	0	45	13	29
Molva molva	1	1	1	0	0	3	1	5	1	2	0	10	3	1	0	75	35	55
Lota lota	1	1	1	0	0	1	1	5	1	2	0	5	3	3	0	78	40	59
Brosme brosme	1	1	1	0	0	1	1	11	1	2	0	9	4	1	0	83	37	60

Merluccius merluccius	1	1	1	0	0	2	1	4	1	2	0	4	5	1	0	76	18	47
Merluccius capensis	1	1	1	0	0	1	4	5	1	2	0	6	4	2	0	-11	-37	-24
Merluccius polli	1	1	1	0	0	1	1	3	1	2	0	5	6	1	0	29	-19	5
Melanonus zugmayeri	1	1	1	0	0	1	1	1	1	1	0	13	6	1	0	60	-49	5.5
Macrourus berglax	1	1	1	0	0	1	2	1	1	1	0	16	3	1	0	82	37	59.5
Malacocephalus occidentalis	1	1	1	0	0	2	1	1	1	1	0	8	2	1	0	43	-37	3
Bathygadus melanobranchus	1	1	1	0	0	1	1	1	1	1	0	2	2	1	0	53	-34	9.5
Muraenolepis marmoratus	1	1	1	0	0	1	3	1	1	1	0	4	5	1	0	-44	-56	-50
Bregmaceros cantori	0	0	1	0	0	1	1	3	1	1	0	0	0	1	0			
Mora moro	1	1	1	0	0	1	1	1	1	1	0	1	1	1	0	64	-51	6.5
Laemonema laureysi	1	1	1	0	0	1	1	4	1	1	0	1	1	1	0	8	-8	0
Polymixia japonica	1	1	1	1	0	1	1	1	1	1	1	2	2	1	0	40	6	23
Percopsis transmontana	1	1	1	0	0	1	1	1	1	1	0	1	0	0	0	44	43	43.5
Typhlichthys subterraneus	1	1	1	0	0	1	1	1	1	1	0	1	0	0	0	39	34	36.5

Zeus faber	1	1	1	0	0	1	1	1	1	1	0	0	0	1	1	75	-49	13
Cyttopsis roseus	1	1	1	0	0	1	1	1	1	1	0	0	0	1	1			
Lamprogrammus exutus	1	1	1	0	1	1	1	1	1	1	1	0	2	0	0	12	-23	-5.5
Brotula barbata	1	1	1	0	1	1	1	1	1	1	1	0	2	1	0	30	-14	8
Carapus acus	1	1	1	0	1	1	1	1	1	1	1	1	1	0	0	42	-15	13.5
Myripristis jacobus	1	1	1	1	1	1	1	1	1	1	0	1	1	0	0	37	-23	7
Holocentrus rufus	1	1	1	1	1	1	1	1	1	1	1	1	3	0	0	33		33
Trachyrincus scabrus	1	1	1	0	0	7	1	1	1	1	0	1	2	1	0	55	-27	14
Chatrabus melanurus	1	1	1	0	1	1	1	1	1	1	0	0	1	1	0	-35		-35
Parasudis fraserbrunneri	1	1	1	0	1	1	1	1	1	1	1	0	1	1	1	21		21
Regalecus glesne	1	1	1	0	0	1	1	1	1	1	0	1	0	0	0	72	-52	10
Lampris guttatus	1	1	1	0	0	1	1	1	1	1	0	1	0	0	0	70	-45	12.5
Guentherus altivela	0	1	1	1	1	1	0	1	1	1	0	1	0	1	1			
Antennarius striatus	1	1	1	0	1	1	1	1	1	1	0	0	1	0	0	43	-50	-3.5

Osmerus eperlanus	1	1	1	0	1	1	1	1	1	1	1	0	0	1	1	70	43	56.5
Perca fluviatilis	1	1	1	0	1	1	1	1	1	1	1	0	17	1	0	74	38	56
Sebastes norvegicus	1	1	1	0	1	1	1	1	1	1	1	1	0	1	0	79	38	58.5
Chaenocephalus aceratus	1	1	1	0	1	1	1	1	1	1	1	3	1	1	0	-53	-65	-59
Borostomias antarcticus	1	1	1	0	1	1	1	1	1	1	1	4	1	1	0	66	-66	0
Benthosema glaciale	0	0	1	0	1	1	1	2	1	0	0	49	8	4	0	81	11	46
Rondeletia loricata	1	1	1	1	1	1	1	1	1	1	0	2	3	1	1	67	-42	12.5
Beryx splendens	1	1	1	1	1	1	1	1	1	1	0	1	1	1	1	45	-43	1
Neoniphon sammara	1	1	1	1	1	1	1	1	1	1	1	1	1	0	0	30	-30	0
Monocentris japonica	1	1	1	1	1	1	1	1	1	1	1	1	1	1	0			
Acanthochaenus luetkenii	1	1	1	0	1	1	1	1	1	2	0	1	0	0	0	40	-57	-8.5
Stylephorus chordatus	1	0	1	0	0	1	1	2	1	1	0	1	0	1	0	45	-37	4
Spondyliosoma cantharus	1	1	1	0	1	1	1	1	1	1	1	1	2	0	0	63	-20	21.5
Thunnus albacares	1	1	1	0	1	1	1	1	1	1	1	0	7	1	0	52	-45	3.5

Helostoma temminckii	1	1	1	0	1	1	1	1	1	1	1	3	14	1	0	16	-6	5
Anabas testudineus	1	1	1	0	1	1	1	1	1	1	1	2	3	0	0	28	-10	9
Selene dorsalis	1	1	1	0	1	1	1	1	1	1	0	2	0	0	0	39	-28	5.5
Chromis chromis	1	1	1	0	1	1	1	1	1	1	1	2	2	1	0	46	-12	17
Parablennius parvicornis	1	1	1	0	1	1	1	1	1	1	0	1	1	1	0	36	-6	15
Symphodus melops	1	1	1	0	1	1	1	1	1	1	1	1	1	0	0	63	28	45.5
Pseudochromis fuscus	1	1	1	0	0	1	1	1	1	1	1	1	1	1	0	26	-24	1
Myoxocephalus scorpius	1	1	1	0	1	1	1	1	2	1	1	2	4	0	0	80	40	60
Trachyrincus murrayi	1	1	1	0	0	3	1	1	1	1	0	3	2	1	0			
Phycis blennoides	1	1	1	0	0	1	1	7	1	2	0	5	1	1	0	71	20	45.5
Lesueurigobius cf sanzoi	1	1	1	0	1	1	1	1	1	1	0	3	7	2	0	42	-21	10.5
Gadus morhua	0	0	1	0	0	4	8	5	1	1	0	12	1	6	0	83	35	59
Astyanax mexicanus	1	1	1	0	1	1	2	1	1	1	0	0	3	0	1	36	24	30
Danio rerio	1	1	1	3	2	1	2	1	1	1	0	0	0	0	4	33	8	20.5

Gasterosteus aculeatus	1	1	1	0	1	1	1	1	1	2	1	1	0	0	0	71	26	48.5
Oreochromis niloticus	0	1	1	0	1	1	1	1	1	1	0	1	2	1	0	32	10	21
Oryzias latipes	1	1	1	0	1	1	1	1	1	1	0	1	0	1	0	55	10	32.5
Poecilia formosa	1	1	1	0	1	1	1	2	1	1	0	1	3	0	0	27	25	26
Takifugu rubripes	1	1	1	0	1	1	1	1	1	1	0	1	1	0	0	46	21	33.5
Tetraodon nigroviridis	1	1	1	0	1	1	1	1	1	1	0	1	1	0	0			
Xiphophorus maculatus	1	2	1	0	1	1	1	1	1	1	0	2	1	0	0	23	17	20
Salmo salar	2	2	2	0	2	4	4	2	2	2	0	2	2	0	4	72	37	54.5

Supplementary table 2 All query TIR domain sequences used for teleost *TLR* characterization derived from Ensembl.org v83. and GenBank.

Species	Fasta sequence
Danio rerio	>1_DARE_ENSDARP00000139386<br>KLTRGMDSILLILLEPLPLYLIPSKYYQLKTMMSRRTYLEWPQEGAKQKLFWANLRAALQAELPNTPDREEE
	>14_DARE_ENSDARP00000106955<br>AIGKTFSDVILVVKEPIDPTSLPSKFCKLKRMLNTKTYLEWPQQPTEQNFFWIQLRSVLGKPNSIRPRTISRHSRLSS<br>ARSVSLIEAPQIQDPEGPDEEDHQNSPQPSNKCQLTCIEVA
	>2_DARE_ENSDARP00000110559<br>IMDEHNDSAVLVLLEPIKKETIPKRFCKLRKIMNSRTYLEWPEDEDKRDEFWSNLRAALQRDEC
	>21_DARE_ENSDARP00000098992<br>LFQEMQDVLLLVFLEPIPERQLSAYHRMRKVMLKKTYLQWPGSNCSDPNSAKELFWNQLKRALRSSNSGSQDE<br>QKMDDNELRRKEKVCADKEEREYFVNQTPTEDEIYYLMP
	>26a_DARE_ENSDARP00000108970<br>LQVEHRDILILVFLETIPSRLLSSHHRLARLVKTRTYLDWPQEPEMHEAFWDRLWCKLSSNKAK
	>26b_DARE_ENSDARP00000115820<br>QLEEQKHRLILIFLQHISPFELSAFHRLAKLVRSRTYLDWPEEEGDREHFWDRL
	>3_DARE_ENSDARP00000014779<br>VMEDNRDSLILIFLEDVTDYNLNRSLHLRRGMLKPKCVLYWPLHKERIPAFHQKLRSALASTNKVN
	>4_a_DARE_ENSDARP00000110093<br>FLMERNANIIIIILEDVAERKTKKILGLHKHLKKNTYLKWSRDPLSNMRFWIRLRKAIVATKQ
	>4_b_DARE_ENSDARP00000028819<br>FLMERNANIIIIILEDVAERKTKKVFGLHKHLKKNTYLKWSRDPLSNMRFWIRLRKAILQK
	>4al_DARE_ENSDARP00000123702<br>FVVERNANIIIIILEDVAERKTKKVLGLHKHLKKNTYLKWSRDPLSNMRFWIRLRKAIVAT
	>5_a_DARE_ENSDARP00000129438<br>MLVELKDILVVLVVGNIPQYRLLKYEQLRSFIENRSYLVWPDDGQDLEWFYDQLLHKIIRNTKVKQTNIKEKDKVD<br>ENNPEAADVQADTAV
	>5_b_DARE_ENSDARP00000124387<br>MQAELEDILVVLVVGNIPQYRLLKYKQVRSFIENRSYLVWPDDCQDLEWFYDQLLHKIRKDIKINQTTKEIKREEA<br>NFNTNTAV
	>7_DARE_ENSDARP00000105671<br>LMDERNDVIVLIFLERMPCHSKYLRLRKRLYKKSVLEWPRNPQAQRYFWFSLRSLMATESQYNTLFQETL
	>8_a_DARE_ENSDARP00000133386<br>LLEENEDVIVLLLLEPVLQYSHFVRLRRRLCARSILEWPHSSSAEAWFWQSLRNAIRVDNQALYSELYSRYFTTK
	>8_b_DARE_ENSDARP00000107821<br>LLEENEDVIVLLLLEPVLQHSHFVRLRRRLCARSILEWPHSSSAEAWFWQSLRNAIRVDNQALYSELYSRYF
	>9_DARE_ENSDARP00000105677<br>LLDEKVDVAVLVLLDFLFPKFKYLQMRKRLCKKSVLSWPRNPRVQPLFWNDLRVALVSDNVRAYNKNVTESFF
Gasterosteus aculeatus	>6_GAAC_ENSGACP00000023718<br>RLAWGTDSVVLVLLEPLPQYLIPSKYYQLKSMMGRHTYLEWPQDRAKHRLFWANLRAALQAELPNAQVTELEE
	>14_GAAC_ENSGACP00000002280<br>AVGKTFSDVILVVKEPMDPNSLPSKYLKLKKMLRTKTYLEWPQQVNQQAFFWTQLKGVLGKPTATQKRTHSVK<br>S
	>2_GAAC_ENSGACP00000024681<br>LFDGSAGGEAAILVLLEPLSKEDVPKRFCKLRKLMSSNTYLEWPQREEGRAEFWRRLRCAVGGGEDDPLP
	>21_a_GAAC_ENSGACP00000012342<br>LFDEHRDVLLLVFLEEISQRQVSSYHRMRKVMLKKTYLQWPGSDCTNPTQAQELFWNKLRRAMRS
	>21_b_GAAC_ENSGACP00000011097<br>LLYDGSDVLLLVFLEEIPERCLTPYTRLRKIVHKKTYLLWPETPQEQDSFWVRLIDALSVTQMQKVVTHFNSVD

>22_GAAC_ENSGACP00000007196
LFDERKDVLVLVFLEDIPGAELSPYYRMRKLLKRRTYLSWPRAGQHPNLFWEKLRQALGTKDDPGREELVPTLLD
RQ
>3_GAAC_ENSGACP00000022288
VIEASRDSVVLVFLQDVHDFKLSRSLFLRRGMLRSCCVLDWPVHKERVPAFHQKLLIALGMTNRLQE
>5_a_GAAC_ENSGACP00000005783
MLEELTNVLIVLVVGKKVAHYQLMRYKAVRAFVQRREYLTWPEDPQDLEWFYERLVSQILKDTKLKKVGEEPDR
GPADEDGVQLQAIRAAAV
>7_GAAC_ENSGACP00000005259
LMDEKNDVIVLIFLEKVACNSKYLRLRKRLFKRSVLEWPTNPQAQLYFWFSLRSVLATESHKQYNNLFKETL
>8_GAAC_ENSGACP00000005236
LLDENKDVIVLLMLEPVLQNSHFLRLRKRMCRKSVLEWPKTPAAEPWFWTTLRNVVKVDNQAICNKTYKKYFTV
K
>9_GAAC_ENSGACP00000013418
LLDEKVDAAILVLLDEMFPKLKYLELRKRLCRKSVLSWPRNPRAQPLFWNQMRMALSSDNLRSYDNNMSESFI

Takifugu rubripes

>1_6_TARU_ENSTRUP00000025134
HLALGSDSVVLVLLEPVPQYLIPSKYYQLKFMMARHTYLEWPQDRAKQRLFWANLRAALQADLPHLTVTEIEE
>14_TARU_ENSTRUP00000001044
AMGKTFSDVILVVKEPIDPNSLPSKYCKLKKMLSTKTYLEWPQQVNQQAFFWAQLRSVLGRPTAVTRGRQSVRS
RISSASISVIGPLVDERNPEMDEDRGTEPNYEVIENSLEVSHQRQIPMVAV
>2_TARU_ENSTRUP00000007454
LFDGVAGEPAILILLEPLNKDDIPRRFCKLRKLLSSTTYLEWPHGEEKVGEFWKALRTALRGEEEWEE
>21_TARU_ENSTRUP00000009725
LFDEHRDVLLLVLLEPISERQLSSYHRMRKVMLKKTYLQWPGSECTNPPQAQGLFWSQLRRAI
>22_TARU_ENSTRUP00000015072
LFDEQKDVLILIFLEDIPTRQLSPFYRMKKMLKSKTYLSWPRAEGHPEVFWEKLRQALLSKDMLDLKPLARGISHK
V
>23_TARU_ENSTRUP00000010624
LFDEQKDVLILLFLEEIPAHQLSPYHRMRKLLKRQTYLSWTQAGRHQAGVFWQNVQRALESGDAPHDQVDPLT
GPAEP
>3_TARU_ENSTRUP00000012195
VIEASRDSVVLVFLQDVHDYKLSRTLFLRRGMLRSCCVLYWPVHKERVPAFHQKLLIALGMTNRLQ
>5_a_TARU_ENSTRUP00000011330
MLEELTNILVMVVVGKVAHYQLMRCKAVRVFVQKRQYLTWPEDPQDLDWFYERLVSLLLKDTKEASSESSVPAS
LASCVF
>7_TARU
LMDEKNDVIVLIFLENIPYNSRYLRLRRRLYRRSVLEWPTNPQARPYFWFSVRSVLATESHKQYNNLFKETL
>8_TARU
LLDENVDVIVLLLLEPVLQHSHFLRLRRRLCGESVVDWPRTAAAEPWFWQNLRNVVRVENQVMYNKNYSKYFT
SK
>9_TARU_ENSTRUP00000019170
LLDEKVDTAVLVLLDEMFPKLKYLQLRKRLCRKSVLSWPRNPKAQPLFWNQLRMALSSDNLSFYDNNMSESFI

Tetraodon nigroviridis

>1_6_TENI_ENSTNIP00000017225
HLARSSDSVVLVLLEPVPQYLIPSKYYQLKAMMARHTYLEWPQDKAKQRLFWANLRAALQADLPDLTVS
>2_TENI
LLDGAAQGRTPVLVLLEPLQDGAVPQALLQAAPLLSSTTYLEWPQQEDKVEAF
>21_TENI_ENSTNIP00000011033
LFDEHRDVLLLVFLEAISKKQLSSYHRMRKVMLKKTYLQWPDSECSDPAQAQDLFWSQLRRAIGTSSRIGTEENG
AGVLDSEVPEQSENHQSNENCFLLP
>22_TENI_ENSTNIP00000016626
LFDERKDVLILIFLEDIPISQLSPFYRMKKMLKRKTYLSWPRAEGHPLVFWEKLRQALLEKETVQLR
>23_TENI_ENSTNIP00000008068
LFDEQKDVLILLFLEEIPAHQLSPYHRMRKLLKRQTYLSWAQAGRHRAGVFWQNVQRALECGDAPHDRVDPLM
GPAQP
>3_TENI_ENSTNIP00000009925

VIEASRDSVVLVFLQDVHDHRLSRTLFLRRGMLRSCCVLYWPVHKERVAAFHQKLLIALGMTNRLH

>5_TENI_ENSTNIP00000008738

MLEELTNTLVMLVVGKVAHYQLMRCNAVRAFVQKREYLTWPEDPQDLDWFYQKLISLLLKHTKVKKCAEENPE
PVAQTADRIQLQDI

>7_TENI_ENSTNIP00000016967

LMDEKNDVIVLIFLEKIPSNSKYLRLRRRLYRRSVLEWPTNPQARPYFWFSLRSVLATESHKQYNNLFKETLKGCIT
VPSLLKMCNGIFVFVFLSQ

>8_TENI

LLDENVDVILLLMLEPVLQHSHFLRLRRRLCGESVLDWPRTAAAEPWFWQNLRNLLRVDNQVMYNKNYSKYFT
GK

>9_TENI_ENSTNIP00000018058

LLDEKVDAAVLVLLDETFPKVKYLQLRKRLCRKSVLSWPKNPKAQPLFWNQVRMALSSDNLSFYDNNMSESF

Oreochromis niloticus

>14_ORNI_ENSONIP00000008442

AMGKTFSDVILVVKEPIDPNSLPSKYCKLKKMLSTKTYLEWPQQVNQQAFFWAQLRSVLGKPTTQERANSVKRR
TLSAGRISVIGPPIEERVPEEDKVTVEKDTEPKKEANKEPLNQIPLVAF

>25_ORNI_ENSONIP00000017788

AISVHRDSLVFILLEPIPTNSLPKKYLRLRSLLRQQTYLEWPKDDRKQQTFWASLKSTLHIADKSVVLKDIAVAISDT
VPLVADQM

>2_ORNI_ENSONIP00000017744

LFEGHATGEAAILILLEPLSKDDVPKRFYKLRKLMSSTTYLEWPQEDEKREEFWRSLRNALRG

>21_ORNI_ENSONIP00000025830

LFDEHRDVLLLVFLEPISERQLSSYHRMRKVMLKKTYLQWPGSDCTNPTQAQELFWNQLRRAIKTGTRLETEECS
ATHESKEAEDFKEERSDENFYLQP

>22_ORNI_ENSONIP00000008192

LFDEQTDVLILVFMEEIPSYLLSPYHRMRKMLKKKTYLSWPRAGEHTELFWEKLCQALETREDPEAKTLLLTVPTM
P

>23_a_ORNI_ENSONIP00000014695

LFDEHKDVLIMVFLEDIPAKQLSPYYQMRSLVKKRSYLSWPQAVQHTGVFWQKIQQALKWKINDCIYQSVKQST
D

>23_b_ORNI_ENSONIP00000014696

LFDEKKDVLILLFLEEIPAQQLSPYYRMRKLVKKRTYLSWPRAGQHPGVFWQNVRRALETGNAPNETTNLLTCSL
PP

>3_ORNI_ENSONIP00000012793

VIEESRDSVILVFLQDVHDHKLFHSLFLRRGMLRPRCILDWPVHKERIAAFHQKLLIALGLTNRLKD

>5_a_ORNI_ENSONIP00000001683

MLEELTNVLIMLVVGKPKVAHYQLMKCNAVRAFVQRKEYLTWPEDPQDMEWFYERLISDILKDSKVKKLKENKP
EPGGPEVQPESHNGINAENIKETSM

>7_ORNI_ENSONIP00000025981

LMDEKNDVIVLIFLEKVPCNSKYLRLRKRLYKRSVLEWPTNPQAQPYFWFSLRSVLATEGHKQYNNLFKETL*REQ
YAGKCSFFSLDCFKCTPLYELLYVNTELFYY

>9_ORNI_ENSONIP00000015639

LLDEKVDVAVLVLMDKMFPKLKYLQLRKRLCRKSVLSWPHHPCAQPLFWNQMRMALSSDNLKFYDNNMSESF
I

Oryzias latipes

>6_ORLA_ENSORLP00000005545

GLSLGTDGIVLVLLEPLPQYLIPNKYYQLKSMMLRHTYLEWPQDRAKHRLFWANLRAALQADLPNLSVTD

>14_ORLA_ENSORLP00000019663

AMGKTFSDVILVVKEPIDPSSLPSKYCKLKKMLSTKTYLEWPQQPNQQQFFWAQLRSVLGKPTETQDRPHSIRKR
NSEGRISVIGPPVEKNMPKEDMPDADSKDLEFDEGNGIELSNLRQIPIMVL

>25_ORLA_ENSORLP00000015994

SISTQSDSIVFVLLEPIPPDSLPKRFLRLRSLLKQQTYLEWPSDERKQQVFWASLKSLL

>2_ORLA_ENSORLP00000003159

LFDAGGDAAILILLEPMTKDDVPKRFCKLRKLMSSTTYLEWPGEEAQREEFWRSLRNALRGE

>21_ORLA_ENSORLP00000016852

LFDEHRDVLLLVFLEPISERQLSSYHRMRKVMLKKTYLQWPGPDTTNFLQARELFWSQLRRAIRAGNRLETEDEA
QEHYA

>22_ORLA_ENSORLP00000025294

LFDDQKDVLILIFLEEIPLNQLSPYHRMRRLLKRQTYLSWARAQNPQVFWEKLRRALQSGDDLDEGLFLFVAPSM
W

>3_ORLA_ENSORLP00000010275

VIEASRDSVVLVFLQDVNDYKLSRSLFLRRGMLRPRCILEWPVQRERVPAFHQKLLIAL

>5_a_ORLA_ENSORLP00000020303

MLEELTNILIMLVVEKMAHYQLMKCNAVRAFVRTREYLTWPEDPQDLDWFYEKLSLQILKDTKVKKFALDEHEA
GKLARAEPIPDGLEDIGMDDIRVAGV

>7_ORLA

LMDEKNDVIVLIFLEKVSCNSKYLQLRKRLYRRSVLEWPANPQAQQLFWFSLRSVLATESHKQYNNLFKETL

>8_ORLA

LLDENVDVIVLLMLEPVLQHSHFLRLRKRLCGKSVVEWPRTAAAEPWFWQNLKTVIRKDNQGMYNKTYSKYFT
NE

>9_ORLA_ENSORLP00000011108

LLDEKMDTAILVLLDETFPKLKYLQLRKRLCRKSVLSWPMNPKAQPLFWNQMRVALALDNLKLYDSNMSESFI

Latimeria chalumnae

>1_6_a_LACH_ENSLACP00000011409

LFSENSNNLILILLEPIPQYLIPDKYYKLKSLMAKRTYLEWPKDKNKNPLFWANLRAAIKFNLQDVNKKISSCLA

>1_6_b_LACH_ENSLACP00000017183

TFDEKKDSLILLLLEPIPTNSIPDRFCKLRKLMNKNTYLEWPQDEARQTLFWQRLKAVLDL

>14_LACH_ENSLACP00000020140

AIGQGFEDVILVVKETINPNSLPNKFCKLRKMLSTKTYLEWPAEPNRQPFFWMQLKNVLGK

>2_LACH_ENSLACP00000014305

LFEGNDSAILILLEPIRKETIPKRFCKLRRMMNTKTYLEWPQDEGQQHLFWFKLKLAIKD

>21_a_LACH_ENSLACP00000001202

LFQEKKDVLILVFLEDIPKGELSKYHRMRKVMLKKTYISWPQEEDAQDLFWARIRKALKESNSNPEQDNIQLKD

>21_b_LACH_ENSLACP00000002971

LFDEHNDVLILVFLEDIPDHVLSSYYRLRKIVRKKTYLKWPSDQAGQKLFWAKLKDALKSETNAHKRELPSIEA

>3_LACH_ENSLACP00000012956

AIEQSHDSIILVFLEDIPDYKLNHALCLRRGMFKSRCILSWPAQRERVNAFHQKFKVALGSSNREH

>5_LACH_ENSLACP00000017461

YFHDCKDVLIVVVVGRIPQYHLMKYKPIRAFMQSRPYLRWPEELQDVDWFLEKLAYLILKKKTVKRKTNNIRLQRI
KTVT

>13_a_LACH_ENSLACP00000007185

LLFDHSDVLILIFLEHIPDYRLSSYYRLCKLVKKTFIQWPEKEEEQELFWMRLKEALRPESVAPEEPKLID

>13_b_LACH_ENSLACP00000014614

LFYNNHDAVILIFLEAIPDYRLSAYQKLRKLVKKQTFISWPEDPNEKQLFWARVRNAIAQKTVNEPNLKLNIAE

>7_a_LACH_ENSLACP00000001158

LIDEKVDVIVLVFLEKALQKSKYLKLRKRLCSSSVLDWPTNPQAQHYFWQCLKNALATDNRMQYSKLFQETL

>7_b_LACH_ENSLACP00000004758

LIDEKLDVIVLVFLEKALQKSKYLKLRKRLCSSSVLDWPTNPQAQRYFWQCLKNALATDNRMQYSKLFQETL

>8_LACH_ENSLACP00000018907

LMDENMDVIVLIVLEPVLQHSQYLRLRRRLCGSSILDWPINPDAERFFWQSLRNVIATDNTVRYNKMYKESIL

>9_LACH_ENSLACP00000020965

LLEEKKDVVILILLDEVLKKSRYLQLRKRLCKKTVLFWPPNPFGQQYFWHKLRTVLDKDNHQYYDKNFSVGFD

Lepisosteus oculatus

>1_6_a_LEOC_ENSLOCP00000015894

LLSQDSDNIILVLLEPVPQYLIPSKFYKLKAMMAKRTYMEWPQDRNKHKLFWANLRAALQVNLSAGVGTPDTE

>1_6_b_LEOC_ENSLOCP00000021836

VFEERRDSLILLLLEPIPANSIPDRFCKLRKMMNKSTYLEWPPDEACQSMFWKRLKAVLNLEVK

>14_LEOC_ENSLOCP00000009707

AIGKTFQDVILVVKEAIDPDSLPSKYCRLKKMLSTKTYLEWPEETQRRAFFWAQLCSVLGKPTLRV

>25_a_LEOC_ENSLOCP00000020879

ALSIQQDSLVFILLEPIPADSLPRKFLKLRALLRKQTYLEWPKEVRKQTFFWASLKGML

>25_b_LEOC_ENSLOCP00000022014

ALTIHQDSLVFILLEPIPADSLPRRFLKLRTLLRQQTYLEWPKEEKKRQFFWTNLKAMLKTANKHMVMKDVAKDI
VDSCPLLKCAE

>2_LEOC_ENSLOCP00000022321

LFDENNDTAILILLEPIPKETIPKRFCKLRKLMNSQTYLEWPEDEALQPIFWHNLKLAIKREEFNLR

>23_LEOC_ENSLOCP00000022417

LFDEQKDVLILVFLEDIPACQLSPYHRMRKLVKKRTYLKWPGHREETRMFWHKLNIALQTRGEADEENPLLSGLD
TQD

>3_LEOC_ENSLOCP00000017046

VIEESRDSIVLIFLDDIPDHKLNHCLYIRRGMLNSRCILNWPLQRTRIPAFHQHLKVALGLSNRVH

>5_LEOC_ENSLOCP00000022101

LFTELQDVLIMVVVGGLPQFKLMKCDPIRTFIKSRHCLHWPEDFQDIDWFYEKLAQNILKEEKSKKQTDVLLQNIG
VQS

>7_LEOC_ENSLOCP00000012197

LMDEKADVIILIFLEKCLQYSKYLRLRKRLCRSSVLEWPSNPQAQHYFWLCLRNTMATDGHTQYNNPFEETL

>8_a_LEOC_ENSLOCP00000012206

LLDDNTDVIVLVLLEPVLLHSQFFRLRKRLCRKTILEWPKNPRAEKWFWHCLRNIIRVEHQAVYNKLYSCYFINK

>8_b_LEOC_ENSLOCP00000012217

LLDDNTDVIVLVLLEPVLKHSQFFRLRKRLCRKTILEWPTNPHAEKWFWHCLRNIIRVEHQAVYNKLYTSYFTSR

>9_a_LEOC_ENSLOCP00000017493

LLDEKVDVVVLVLLDKVFYKSRYLQMRKILCRKSVLTWPMNPHAHQHFWNSMKTILASDNCKYYDSNVNESFLI
NNLV

>9_b_LEOC_ENSLOCP00000017758

LLEERADVAVFVLLEKGRRGSRYLQLRRLLCRETVLLWPQNPQAQGYFWHRLRCVLGRDIQASYDRKHCRGVPT
PL

Anolis carolinensis

>1_6_ANCA_ENSACAP00000007179

LFSEDVYGLILVLLEPIPTYIIPARYHKLKALMAKRTYLEWPKEKSKHGLFWANLRAAIQTVLPNNEEMEELQG

>1_6b_ANCA_ENSACAP00000019120

LFREDVYGLILVLLEPIPTYIIPARYHKLKALMAKRTYLEWPKEKSKHGLFWANLRAAIQIKLPNNDEMEE

>14_ANCA_ENSACAP00000020761

AVGLGFEDVVLVVKEAVDPQALPRKFCKLRKLLSAKTYLEWPAEPGRQAFFWAQLAAVLGK

>2a_ANCA_ENSACAP00000019306

LFDENNDSAILILLEPIPAETVPRRFCKLRKLMNTKTYLEWPSDEDQKSIFWFNLKTAIKS

>2b_ANCA_ENSACAP00000017757

LFDENNDSAILILLEPIPEETVPRRFCKLRKLMNTKTYLEWPSDEDQKIIFWFNLNIAMKS

>21_ANCA_ENSACAP00000014787

LFDELKDVLVPVFLEEIPERELSAYYRMRKVMLKKTYVTWPPETQAQQLFWAKLRMALAAGTTEEEEDGGRTTH

>23_ANCA_ENSACAP00000018176

IFDQHKDVLILIFLEDIPCEYLSPYHRMRRLIKKKTYLKWPQEEQEIQFFWHKLNMAMKTGEGKEEE

>3_ANCA_ENSACAP00000001660

AIEQSRDSIVLIFRDDIPDYKLNHALCLRRAMFKSCCILEWPVQKDHVNAFYQQLKIALQTSSRIL

>4_ANCA_ENSACAP00000013654

QLLDSKASLVLVVLEGVDNAVVRQKLGLFRYLRRNTYLVWRDRELNRHMFLRQLKSALLEGKTWTEEELKLMLT
N

>5_ANCA_ENSACAP00000002366

YFCDLKDVLIMVVAGSLSQYQLMKYQPVRAFLQRDRYLRWPEEDQDVEWFLNALSHQILKEKRTQKKAQKKKV
PKKTGTLELKVVTIS

>13_ANCA_ENSACAP00000016366

IYYHGQDPLVVVFMEEISNYRLSPYHRLRKLIKQESYLTWPEEPEAEAVFWTRLREALSPGEQEQEFKPFNIVE

>7_ANCA_ENSACAP00000001544

LMDEKVDVIILIFLEKVLQMSKCLRLRKILCSQSVLEWPTNPRSQCYFWHCLRNSLTANHDTSYSKLFKEMV

Xenopus tropicalis

>6a_XETR_ENSXETP00000057556

LFGKNSNNLILILLDPIPQYSIPNKYNKLRSIMKHRTYLEWPKEKGKHALFWANLREAIHVNLSIKEED

>6b_XETR_ENSXETP00000057561

LYTENNDNLILILLEPIPQYLIPSKYYKLKTLMAQRTYLEWPSEKSKHGLFWANLRAAISIDLTHAESE

>14a_XETR_ENSXETP00000054703

AVGHALEDVILVVKEKVTMEDLPKRFQKLRKLLRTKTYLEWPLEHTRQHFFWIQLRSILGKVSSPVIGQDDLLVDNGAAASWDGPASEAHHEDEI

>14b_XETR_ENSXETP00000038393

AIGHSFEDVILVVKENVTLKDLPKRFHKLRKMLRKKTYLEWPSEPSKQHFFWIQLKNILGSPST

>14c_XETR_ENSXETP00000054700

AIGHAFEDVILVVKEKVTMEDLPKRFQKLRKMMRTKTYLEWPSEQNRQHFFWIQLKSILGKANPP

>14d_XETR_ENSXETP00000054707

AIGHSLEDIILVVKEKVTMEDLPKRFHKLRKMLRTKTYLEWPSEPSKQHFFWIQLKSIL

>2a_XETR_ENSXETP00000003822

LFDENNDSAILILLEPIENETVPKRFCKLRKLMNTKTYLEWPTDEEQQEVFWDNLKTALQS

>2b_XETR_ENSXETP00000003835

LFDENNDSAILILLDPIEKETVPKRFCKLRKLMNTKTYLEWPTDEEQQEVFWDNLKTALQPANAHRP

>21_XETR_ENSXETP00000059090

LFDEMRDVLVLILLDNIPDRELSTYHRMRKLMLKKTYITWPTEPEAQQLFWAKVQEAVSGKRPSEGHQGIKIVK

>23_XETR_ENSXETP00000055333

LFEEHTDVLILLFLEQIPDHHLSPYHQIRKVIKKKTYLIWPQERNATSIFWYRVSQALQTEEREEDEK

>26_XETR_ENSXETP00000061916

LLAEKNDDLILVFLESISGYHLSAYHRLARIVRKKTYIDWPQEENEQAEFWERLRMTVL

>3_XETR_ENSXETP00000012947

AIEQNRDSIILIFLEDIPDYKLYHTIHLRRGMFKSRCILDWPDQMERIKTFYQRLKIALGSTNLVN

>5_XETR_ENSXETP00000032872

YFTELKDVLIMVVVGSLSQYQFMKYQPIRAYVKRAQYLRWPEDIQDVEWFLGRLSYQILKENTVEKKPNQASRKPFNHELQNIETIS

>13_XETR_ENSXETP00000009956

AFSYKEDVLLMVFLEEIPEYRLSAYHKLRKLIKQNTYIDWPEDPRGEEVFWLKLRQALDGGVCEENNIQLCD

>7_XETR_ENSXETP00000011725

LIEEKVDVIILILLEKTLQRSRYLRLRKRLCANSVLYWPSNPNSQSYFWHCLKSAIATENQMGYDKLFKDHT

>8_XETR_ENSXETP00000062585

LMDENMDVIVIVLLEPVLQNSQYLRLRRKICKSSIMEWPKNPNTKSFFWQRMKNVLLTDNCNRYNNFYTDTIAN

>9_XETR_ENSXETP00000013094

LLDEKKDVVALVVLDHKMKMSQYFLTRKRLCPKSFLNWPCNPKAHSHFWHMLRIYIRQDSRRCCGSQLKKYV

Poecilia formosa

>1_6_POFO_ENSPFOP00000020173

RLSQGSDSLVLVLLEPLPQYLIPSKYYQLKSMMKRHTYLEWPQDKAKHRLFWANLRAALQADLPSKLVAETEE

>14_POFO_ENSPFOP00000018414

AMGKTFSDVILVVKEPIDPSSLPSKYCKLKKMLSTKTYLEWPQQVNQQPFFWAQLRSVLGKPTMTREGTNSIRSRNTSERFSVVEERIPEVNKPLEGNKKEIQSEIIHRTEIELQDQRNLEAQ

>2_POFO_ENSPFOP00000000533

LFEAGGDTAILILLEPLSKDDVPKRFCKLRKLMSSTTYLEWPQEEERRPEFWRSLRNALRGDNEQEN

>21_POFO_ENSPFOP00000020442

LFDEHRDVLLLVFLEPISERQLSSYHRMRKVMLKKTYLQWPGPDCTDPSQAQELFWNQLRRAIRAGNRLETEQNDPDEDFARASNETGNYEKLLSDDEDYLQP

>22_POFO_ENSPFOP00000004631

LFDEQKDVLILVFLEDIPTYLLSPFHRMRKLLKKQTYLSWPRAADHPEVFWENLRKALQTGNDPNEEKLQLAVIETY

>23_a_POFO_ENSPFOP00000024685

LFDEKKDVLILLFLEEIPSYHLAPYYRMRKLVKKRTYLSWPQAAQHPGVFWQNVQRALQAGGALTENTDPLTGPAGI

>23_b_POFO_ENSPFOP00000028752

LFDEKKDVLILLFLEDIPPQHLSPYHRMRKLLKKCTYLSWSKAEKHPGVFWQNVRKALQTKDDPPKNPALLTGPTQC

>23_c_POFO_ENSPFOP00000011089

LFDEQKDVLVLLFLENIPAHHLSPYYRMRKLVKKRTYLSWPQAAQHPGVFWQNVQRALQAGGALAENTDLLLGPAGR

>3_POFO_ENSPFOP00000005811

VIEASRDSVVLIFLQDVHDYKLSRSLFLRRGMLHQRCVLNWPAQKERIPAFNQKLLIALGMTNRLQE

>5_POFO_ENSPFOP00000000698
MLEELTNVLVMLVVGKVAHYQLMKCNAVRAFVQRREYLTWPEDPQDLEWFYERLVSLILRDTKVKKFAEDKPE
TVKPDCQPPAKEKIPLENIRVNNLAGNPLM
>7_POFO_ENSPFOP00000025729
LMDEKNDVIVLIFLEKVSCNSKYLRLRKRLYKRSVLEWPTNPQAQPYFWFSLRSVLATESHKQYSNLFKETL
>8_POFO_ENSPFOP00000027487
LLDENVDVIVLLMLEPVLQHSHFLRLRRRLCEKSVVEWPRTAAAEPWFWQNLRSVVKVDNQIMYSKTYKKFFSS
K
>9_POFO_ENSPFOP00000000377
LLDEKVDAAVVVLLDEMFPKLKYLQLRKRLCRKSVLSWPRNPRAQPLFWNQMRMALSSDNLKFYDNNMSESFI

Astyanax mexicanus

>1_6_ASME_ENSAMXP00000026268
RLTRGLDSIILILLEPLPPYLIPSKYHHLKAMMARRTYLEWPQDKTKQRMFWANLRAVLQADLPRSSSLEAGNQE
>14_ASME_ENSAMXP00000019326
AIGKNLRDVILVVKEPIDPKSLPSKYCKLKKMLNTKTYLEWPEQPKQQAFFWAQLRSVMGKPNLLKQNSVIENRR
VSKLSRASVIELPEVKPQITAGTEELPERNTENCSLRMPLEQLA
>2_ASME_ENSAMXP00000026287
IVDEHNDSAVLILLEPIAKQTIPKRFCKLRKIMNSKTYLEWPQEEERREEFWSNLQAALKR
>21_ASME_ENSAMXP00000025842
LFHEMQDVLLLVFLESIPERKLSTYHRMRKVMLKKTYLQWPGPDCTDPAKAQGLFWKQLKKALRSSSSTRQEEE
SMEADVQLGEEKDAPNTEDREYFVNQPHVDEEAYYLMP
>23_a_ASME_ENSAMXP00000025763
LFDEQKDVLILVFLEDIPTQQLSPYHQMRKLVKKKTYLKWPKPGEDTKVFWQKLRLALETKEDLDENALL
>23_b_ASME_ENSAMXP00000026712
LFDEKKDVLILVFLEDIPNSELSPYYRMRRLIKKRTYLKWPKLGKDTQYFWQKLRAALETRGSSDENNSALQSAQ
>23_c_ASME_ENSAMXP00000026713
LFDEKKDVLILVFLENIPAHQLSPYYRMRKLIKKRTYLSWPKPGEDTRVFWEKLRVALETKGTYEEESLLLSGIRRT
>26_ASME_ENSAMXP00000026963
LQVEHRDILILVFLEKIPSKMLSAHHRLARLVKTRTYLAWPQKAELQDEFWERM
>5_ASME_ENSAMXP00000026717
MLEEVQDILLMLLVDQIPQYRLMKYEPIRTYVQTKRYLCWPEDSQDLQWFYNQLQHSILKGTKAKQAKIDAETA
NNVPEVEAVTVF
>7_ASME_ENSAMXP00000026765
LLDEKNDVIILIFLEKFSSSHSKYLRLRKRLYKRSVLEWPRNPRAQPYFWFCLKSVMAAESQQHYNKLFQETL
>8_a_ASME_ENSAMXP00000026319
LLEEGVDVMVLILLEPVLQHSRILHLRRCLCGNSVLEWPRNPSAQNWFWQSLRNAIKKEKQGMQNKLFKNY
>8_b_ASME_ENSAMXP00000001765
LLEESKDVIVLLMLEPVLQSSHFLRLRQRLCSQSVLEWPRAPAAEPWFWQCLRNAIRVENCDMYSKIYSRYFTSRS
SSVRD
>9_ASME_ENSAMXP00000027050
LLDEKVDVAVLVLLDSVFPKVKYLQMRKRLCKKSVLSWPKNPQAQPLFWNDLCVAIASDNVKSYDKKVTESFLSH
ELL

Gadus morhua

>14_GAMO_ENSGMOP00000004019
AMGKAFSDVILVVMEPIDPHSLPSKYCKLKKMLSTKTYLEWPQQPKHQPFFWAQLKSVLGKPSLTRGRAGSTRS
RAASEGRVSMIEPPMEVDQQDHGVNKAELHGEEIETKELFLKPIPIAV
>25a_GAMO
AIDAQQDSLVFILLEPIPTNSLPKKFLKLRRLLMQQTYLEWPKDERKQQVFWVNLRSMLQVADHRILKDMALELT
QSASLIPDDLVPLLK
>25c_GAMO
ALDAQQDSLVFILLEPISTNSLPKKFLKLRRLLMQQTYLEWPKDERKQQVFWVNLRTMLQVADHRILKDMALELT
QSASLIPDDLDPLLK
>25d_GAMO
AIDAQQDSLVFILLEPIPTNSLPKKFLKLRRLLMQQTYLEWPMDERKQQVFWVNLRSMLQVADHRILKDMALEL
TQSASLIPDDLVPLLK
>25e_GAMO
AIDAQQDSLVFILLEPIPTNSLPKKFLKLRRLLMQQTYLEWPMDERKQQVFWVNLRSMLQMADHRILKDMALEL
TQSASLIPDDLVPLLK

>25f_GAMO

AIDAQQDSLVFILLEPIPTNSLPKKFLKLRKLLMQQTYLEWPKDERKQQVFWVNLRSMLQVADHRILKDMALELTQSASLIPHDLVPLLK

>21_GAMO_ENSGMOP00000019623

LFDEHRDVLLLVFLEHIPERELSSYHRMRKVMLRKTYLQWPGSECTDPAMAQLLFWKQLRRALRTGSRMEDEDAAGEKKSSGTAEGDQTDSAVNPTAEEIYQ

>22a_GAMO

LFDEQKDVLILVFLEDIPMQQLSPYYRMRRLLKRKTYLSWSRADAHPDLFWEKLRQALDTQEHPMGEHLRLTVEDGPTGERPDQ

>22b_GAMO

LFDEQKDVLILVFLEDIPMQQLSPYYRMRRLLKRKTYLSWSRADAHPDLFWEKLRQALETQEHPMGEHLRLTIEDGPPGERQDQS

>22c_GAMO

LFDEQKDVLILVFLEDIPMQQLSPYYRMRRLLKRKTYLSWSRADAHPDLFWEKLRQALDTQEHPMGEHLRLTVEDGPPGERPDQ

>22d_GAMO

LFDEQKDVLILVFLEDIPMQQLSPYYRMRRLLKRQTYLSWSRADAHPDLFWEKLRQALETREHPMGEHIRLTVVNGPPGERPDH

>22e_GAMO

LFDEQKDVLILVFLEDIPMQQLSPYYRMRRLLKRQTYLSWSRADAHPDLFWEKLRQALDTQEHPMGEHLRLTVEEGPPGERPDQ

>22f_GAMO

LFDEQKDVLILGFLEDIPMQQQLSPYYRMRRLLKRQTYLSWSRADAHPNLFWEKLRQALETQEHPMGEHILLTVEDGPPGERPDQ

>22g_GAMO

LFDEQKDVLILVFLEDIPMQQLSPYYRMRRLLKRQTYLSWSRADAHPNLFWEKLRQALETQEHPMGEHILLTVEDGPPGERPDQ

>22h_GAMO

LFDEQKDVLILVFLEDIPMQQLSPYYRMRRLLKRQTYLSWSRADAHPNLFWEKLRQALETQEHPMGEHILLTVEDGPPGERPDQ

>22j_GAMO

LFDEQKDVLILVFLEDIPMQQLSPYYRMRRLLKRQTYLSWSRADAHPDLFWEMLRQALETREHPMGEHIRLTVVNGPPGERPDH

>22l_GAMO

LFDEQKDVLILVFLEDIPMQQLSPYYRMRWLLKRQTYLSWSRADAHPDLFWEKLQQALDTQEHPMGEHLRLTVVGSPGERPDQ

>23_GAMO

LLDEKKDVLILLFLEELPEKQLSPYYRMRKLVRRSTYLSWPQAARHPGLFWQNVHRALESGDSHDDNTHFLSGPAL

>3_GAMO_ENSGMOP00000000792

VMEERRDAVILVFLQDINDYRLSRSLFLRRGMLQLRCLLNWPVHKERIPAFHEKLHIALGTTNTPKP

>7a_GAMO

LMDEKDDVIVLIFLEKAASNSKYLRLRKRLYRRSVLEWPTNPQAQPYFWFGLRSVLATESNKQYSDMFRETL

>7b_GAMO

LMDEKDDVIVLIFLEKAASNSKYLRLRKRLYRRSVLEWPTNPQAQPYFWFGLRSVLATESNKQYYSDMFRETL

>8a_GAMO

LLDENLDVIVVLMLEPVLQNSHFLRLRRRLCGESVVEWPRTAAAEPWFWQNLRNVVRVDNQTMYTSTYSQYFTCSRERD

>8c_GAMO

LLDENLDVIVVLMLEPVLQNSHFLRLRRRLCGESVVEWPRTAAAEPWFWQNLRNVVRVDNQTMYTSTYSQYFTCSRERD

>8d_GAMO

LLDENLDVIVVLMLEPVLQNSHFLRLRRRLCGESVVEWPRTAAAEAWFWQNLRNVVRLDNQTMYTSTYSQYFTCSRERD

>8e_GAMO

LLDENLDVIVVLMLEPVLQNSHFLRLRRRLCGESVVEWPRTAAAEPWFWQNLRNVVRVDNQTMYTSTYSQYFTCSRERD

>8f_GAMO

LLDENLDVIVVLMLEPVLQNSHFLRLRRRLCGESVLEWPRTAAAEPWFWQNLRNVVRVDNQTMYPSTYSQYFTCSRERD

>8j_GAMO

LLDENLDVIVVLMLEPVLQHSHFLRLRRRLCGESVVEWPRTAAAEPWFWQNLRNVVRVDNQTMYTSTYSQYFT
CSRRERD

>8l_GAMO

LLDENLDVIVVLMLEPVLQNSHFLRLRRRLCGESVVEWPRTVAAEPWFWQNLRNVVRVDNQTMYTSTYSQYFT
CSRERD

>9a_GAMO

LLDEKVDVAVLILLDKMFPKLKYVQLRTRLCKKSVMSWPRNPKAQPLFWNQMRIALSSDNLKLYDKNISESFI

>9b_GAMO

LLDEKVDVAVLILLDKMFPKLKYLQLRTRLCKKSVMSWPRNPQAQPLFWNQMRTALSSDNLKLYDSNISESFI

>9c_GAMO

LLDEKVDVAVVILLDKMFPKLKYLQLRTRLCRKSVMSWPRNPKAQPLFWNQIRTALSSDNLQIYDNNFSEGFI

>9d_GAMO

LLDEKVDVAVLILLDKMFPKLKYLQLRTRLCRKSVMSWPRNPQAQPLFWNQMRTALSSDNLKLYDNNISEGFI

>9e_GAMO

LLDEKVDVAVLILLDKMFPKLKYLQLRTRLCRKSVMSWPRNPQAQPLFWNQMRTALSSDNLKLYDNNISESFI

Xiphophorus maculatus

>1_6_XIMA

RLSQGSDSIVLVLLEPLPQYLIPSKYYQLKSMMNRHTYLEWPQDKAKHRLFWANLRAALQTDLPSKLVSETEE

>14_XIMA_ENSXMAP00000013389

AMGKTFSDVILVLKEPIDPSSLPSKYCKLKKMLSTKTYLEWPQQVNQQPFFWAQLRSVLGKPTMTREGTNSIRSR
NSSE

>2_a_XIMA_ENSXMAP00000008276

LFEAGGDAAILILLEPLSKDNVPKRFCKLRKLMSSTTYLEWPQEEERRPEFWRSLRNALRGDND

>2_b_XIMA_ENSXMAP00000008281

LFEAGGDAAILILLEPLSKDDVPKRFCKLRKLMSSTTYLEWPQEEERRPEFWRSLRNALRGDNEQEN

>22a_XIMA

LFDEQKDVLILVFLEDIPTYLLSPFHRMRQLLKKQTYLSWPRAAGHPEVFWENLRKALQTGNNANEENFLSLTQE

>22b_XIMA

LFDEQKDVLILVFLEDIPTYLLSPFHRMRKLLKKQTYLSWPRAAGHPEVFWENLRKALQTGNDPNEEKLQLTVTET
Y

>23_XIMA

LFDEKKDVLILLFLEEISAHHLSPYYRMRKMLKKRTYLSWSAQHPGVFWQNVQRALQAGDAPENSDMLTGPTG
R

>3_XIMA_ENSXMAP00000018909

VIEASRDSVVLIFLQDVHDYKLSRSLFLRRGMLRPSCVLNWPPQKERIPAFNQKLLIALGMTNRLQE

>5_XIMA_ENSXMAP00000001784

MLEELTNVLVMLVVGKVAHYQLMKCNAVRAFVQRREYLTWPEDPQDLEWFYERLVSLILRDTKVKKFVEDKPEF
VKPDCQPPAKDKIPLENIGVVANSDVGNDVNELTFINVKEEGLYEPTKADSTHSAQVIVTSP

>7_XIMA_ENSXMAP00000004436

LMDEKNDVIVLIFLEKVSCNSKYLRLRKRLYKRSVLEWPTNPQAQPYFWFSLRSVLATESHKQYSSLFKETL

>8_XIMA_ENSXMAP00000004435

LLDENEDVIVLLMLEPVLQHSHFLRLRRRLCEKSVVEWPRTPAAEPWFWQNLRSVVKVDNQIMYSKTYKKFFSSK

>9_XIMA_ENSXMAP00000007651

LLDEKVDAAVVVLLDEMFPKLKYLQLRKRLCRKSVLSWPRNPRAQPLFWNQMRMALSSDNLKFYDNNMSESFI

Petromyzon marinus

>1a_PEMA_ENSPMAP00000011399

LMESRDGALVLVLLEPIPRDSVPSRFCRLRRLMARKTYLEWPVEQSKQALFWANLRATLSCQREQEPQNLEIVV

>1b_PEMA_ENSPMAP00000011400

LMESRDDALVLVLLEPIPRDSVPSRFCRLRRLMGRKTYLEWPAEQGKQVLFWANLRATLGE

>25a_PEMA_ENSPMAP00000011066

VLEQRQDSLVLLLLEPLPRNSVPSKFCRLRKLLNRKTYLEWPAEEGKRSMFWASLRAVLQSDHEPSNPNSNHRSIS
TCNSNPIRNPYV

>25b_PEMA_ENSPMAP00000011211

MVEENQDSLVLLLLEPLPKGSLPSKFCKLRRLLSNKTYLEWPTEERKRAVFWTSLNAVLQSRPPP

>21a_PEMA_ENSPMAP00000011080

LFDEHKDVVIMVFLEDIPDYKLSAYHRMRKIIGDKTYISWPEDAAGKDLFWAKLRAAI

>21b_PEMA_ENSPMAP00000011162

LFNEHKDVLIMVFLETIPSYKLSAYYRMRRIVNNKTYIVWPDDNIGRELFWAKLRTAL

>21c_PEMA_ENSPMAP00000005491

LFSEHRDVLVTLIVEDIADRELSAHHRMHRLLCTKTHVVWPRDEAARPLFWAKLRTALGEPSAAA

>3_PEMA_ENSPMAP00000001776

AIEQNRDDVVLALLEDIPEFELHHKLCLRAAMFPRRCVLRWPQDPTRLPLFLQQLRVALGS

>7_8_9a_PEMA_ENSPMAP00000002547

LLDEKVDMVILVMLERMRSSFTRSRYMRLRQRLSPGSILRWPTNPHAQKIFWQGLRDALAGPSKAATQAAKRR
R

>7_8_9b_PEMA_ENSPMAP00000006887

MIDEKKDVMVFVMLERMPSRFLYSRYMRMRKRLCPDSFLQWPPNPHAQHLFWKCLRAEITKSQTEQYWQVY
EITV

Gallus gallus

>1_GAGA

LFSENSNSLILILLEPIPSYVIPARYHKLKALMAKRTYLEWPKERSKHALFWANLRAVVNIKLPTSFETDEEQSDVTST
SSITQCLIK

>6_GAGA_ENSGALP00000028126

LFSENSNSLILILLEPIPPYVIPARYHKLKALMAKRTYLEWPKERSKHALFWANLRAAVNIKLPTSFETDEEQSDVTS
TSSITQCLIK

>15_GAGA_ENSGALP00000013260

VLDENQDSLIMVVLEDLPPDSVPQKFSKLRKLLKRKTYLKWSPEEHKQKIFWHQLAAVLKTTNEPLVRAENGPNE
DVIEME

>16_GAGA_ENSGALP00000043467

LFSENSNSLILILLEPIPPYVIPARYHKLKALMAKRTYLEWPKERSKHALFWANLRAAISINLSVADEQNRTEV

>2a_GAGA

LFDENNDVAILILLEPIQSQAIPKRFCKLRKIMNTKTYLEWPPDEEQQQMFWENLKAALKS

>2b_GAGA

LFDENNDVAILILLEPIQSQAIPKRFCKLRKIMNTKTYLEWPPDEEQQQMFWENLKAALKS

>21_GAGA_ENSGALP00000001118

LLDERRDILVLVLLEDVGDAELSAYHRMRRVLLRRTYLRWPLDPAAQPLFWARLKRALRWGEGGEEEEEEGLGG
GTGRPREGDKQM

>3_GAGA_ENSGALP00000021914

AIEQSRDSIILIFLHNIQDYKLNHALCLRRGMFRSCCILNWPVQKERINAFHQQLMMALKSNSK

>4_GAGA_ENSGALP00000011319

QLVEGKAGIIMIILGEVDKTLLRQRLGLSRYLRRNTYLEWKNKEISRHIFWRQLTSVLLEGKKWNHEEIKLM

>5_GAGA_ENSGALP00000015286

YFSDLKEVLIMVVVGSLSQYQLMKHKPIRIFLQRSRYLRWPEDYQDIGWFLDNLSSQILKEKKVQRNVSGIELQTIA
TVSH

>7_GAGA_ENSGALP00000026726

LLDEKIDVIILIFLEKVLQKSRYVQLRKRLCRSSVLEWPTNPRSQPYFWQRLKNAIAMNNTLSYNKLLQETV

Homo sapiens

>1_HOSA_ENSP00000354932

LFHEGSNSLILILLEPIPQYSIPSSYHKLKSLMARRTYLEWPKEKSKRGLFWANLRAAINIKLTEQAKK

>6_HOSA_ENSP00000480266

LFHEGSNNLILILLEPIPQNSIPNKYHKLKALMTQRTYLQWPKEKSKRGLFWANIRAAFNMKLTLVTENNDVKS

>10_HOSA_ENSP00000478985

LFHENSDHIILILLEPIPFYCIPTRYHKLKALLEKKAYLEWPKDRRKCGLFWANLRAAINVNVLATREMYELQTFTEL
NEESRGSTISLMRTDCL

>2_HOSA_ENSP00000260010

LFDENNDAAILILLEPIEKKAIPQRFCKLRKIMNTKTYLEWPMDEAQREGFWVNLRAAIKS

>3_HOSA_ENSP00000423684

AIEQNLDSIILVFLEEIPDYKLNHALCLRRGMFKSHCILNWPVQKERIGAFRHKLQVALGSKNSVH

>4_HOSA_ENSP00000377997

QFLSSRAGIIFIVLQKVEKTLLRQQVELYRLLSRNTYLEWEDSVLGRHIFWRRLRKALLDGKSWNPEGTVGTGCN
WQEATSI

>5_HOSA_ENSP00000440643

CLSDLNSALIMVVVGSLSQYQLMKHQSIRGFVQKQQYLRWPEDLQDVGWFLHKLSQQILKKEKEKKKDNNIPLQ
TVATIS

>7_HOSA_ENSP00000370034

LMDEKVDVIILIFLEKPFQKSKFLQLRKRLCGSSVLEWPTNPQAHPYFWQCLKNALATDNHVAYSQVFKETV

>8_HOSA_ENSP00000312082

LMDENMDVIIFILLEPVLQHSQYLRLRQRICKSSILQWPDNPKAEGLFWQTLRNVVLTENDSRYNNMYVDSIKQY

>9_HOSA_ENSP00000353874

LLEDRKDVVVLVILSPDGRRSRYVRLRQRLCRQSVLLWPHQPSGQRSFWAQLGMALTRDNHHFYNRNFCQGPT
AE

Mus musculus

>11_MUMU_ENSMUSP00000068906

LVARPGTTHLLLLFLEPLDRQRLHSYHRLSRWLQKEDYFDLSQGKVEWNSFCEQLKRRLSKAGQERD

>12_MUMU_ENSMUSP00000074381

LLAAPSPPVLLLVFLEPISRHQLPGYHRLARLLRRGDYCLWPEEEERKSGFWTWLRSRLG

>13_MUMU_ENSMUSP00000043101

MFYEHKDVIILIFLEEIPNYKLSSYHRLRKLINKQTFITWPDSVHQQPLFWARIRNALGKETVEKENTHLIVVE

Rattus norvegicus

>11_RANO_ENSRNOP00000051449

LIARPGTTCLLLLFLEPLDRQKLHSYHRLSRWLQKEDYFDLSQGKMEWNTFCEQLKQRLRKARKERED

>12_RANO_ENSRNOP00000062596

LLAAPSPPVLLLVFLEPISRHQLPSYHRLARLLRRGDYCLWPEEEERKGGFWTWLRSRLG

Ictalurus punctatus

>25_ICPU_AEI59680.1

AISVRDDSLVFILLEPIPSDSLPKKFLRLSTMLRQKTYLEWPKDXTKKKVFWSSLRSILQTADKRMVLKEIACDIAEN
TLLLNAQQ

>26_ICPU_AEI59681.1

LLVENRDVLIVVFLEKISPRQLSAHHRLARVVKKKTYIDWPEEPGRRTAFWDRLWAKLAPEPARTQR

>21_ICPU_AEI59678.1

LFDEMQDVLLLVFLEDIHERQLSAYHRMRKFMLKKTYLQWPGLDCTDPIKAQELFWTQLKRALRSSNSRSQDEE
QTLENVQNGPQIDDARVEEREYFMNQGQMDDGPYYLTP

SLOUCH results

BEST ESTIMATES & MODEL FIT – TLR8 phylogeny

MODEL PARAMETERS

	Estimate
Rate of adaptation	11.7482573
Phylogenetic half-life	0.0590000
Phylogenetic correction factor	0.9148817
Stationary variance	4.1000000

PRIMARY OPTIMA

	Estimate	Std.error
Theta_global	1.454513	0.2747245

MODEL FIT

	Value
Support	-129.99582
AIC	265.99165
AICc	266.40544
SIC	272.37305
r squared	0.00000
SST	61.78786
SSE	61.78786

> model.fit(ancestor, indata3$time, seq(0.00,0.1, 0.001), seq(1, 10, 0.1), response= indata3$TLR8, me.response= NULL, fixed.fact=NULL, fixed.cov= NULL, me.fixed.cov= NULL, mecov.fixed.cov=NULL, random.cov= NULL, me.random.cov=NULL, mecov.random.cov=NULL, intercept="root", ultrametric=TRUE, support=NULL, convergence=NULL)

BEST ESTIMATES & MODEL FIT – TLR8 group 0

MODEL PARAMETERS

	Estimate
Rate of adaptation	16.9060288
Phylogenetic half-life	0.0410000
Phylogenetic correction factor	0.9408495
Stationary variance	3.2900000

PRIMARY OPTIMA

UNKNOWN mapped to the root of the tree and includes the coefficent for the ancestral state (Ya)

	Estimates	Std.error
UNKNOWN	5.8583640	1.2414273
NO	1.5558946	0.4024705
YES	0.9097707	0.3409476

MODEL FIT

	Value
Support	-123.91445
AIC	257.82890
AICc	258.90033
SIC	268.46457
r squared	20.12795
SST	77.60318
SSE	61.98325

```
> model.fit(ancestor, indata2$time, seq(0.001,0.1, 0.01), seq(3.1, 3.7, 0.01), response= indata2$TLR8,
me.response= NULL, fixed.fact=Group_0, fixed.cov= NULL, me.fixed.cov= NULL,
mecov.fixed.cov=NULL, random.cov= NULL, me.random.cov=NULL, mecov.random.cov=NULL,
intercept="root", ultrametric=TRUE, support=NULL, convergence=NULL)
```

BEST ESTIMATES & MODEL FIT – TLR8 group 25

MODEL PARAMETERS

	Estimate
Rate of adaptation	16.9060288
Phylogenetic half-life	0.0410000
Phylogenetic correction factor	0.9408495
Stationary variance	3.3500000

PRIMARY OPTIMA

UNKNOWN mapped to the root of the tree and includes the coefficent for the ancestral state (Ya)

	Estimates	Std.error
UNKNOWN	5.990984	1.2472106
NO	1.442845	0.4835486
YES	1.072728	0.3106295

MODEL FIT

	Value
Support	-124.45568
AIC	258.91137
AICc	259.98279
SIC	269.54704
r squared	18.72125
SST	76.21327
SSE	61.94520

```
> model.fit(ancestor, indata2$time, seq(0.001,0.1, 0.01), seq(3.3, 3.7, 0.01), response= indata2$TLR8, me.response= NULL, fixed.fact=Group_25, fixed.cov= NULL, me.fixed.cov= NULL, mecov.fixed.cov=NULL, random.cov= NULL, me.random.cov=NULL, mecov.random.cov=NULL, intercept="root", ultrametric=TRUE, support=NULL, convergence=NULL)
```

BEST ESTIMATES & MODEL FIT – TLR8 group 50

MODEL PARAMETERS

	Estimate
Rate of adaptation	16.9060288
Phylogenetic half-life	0.0410000
Phylogenetic correction factor	0.9408495
Stationary variance	3.3300000

PRIMARY OPTIMA

UNKNOWN mapped to the root of the tree and includes the coefficent for the ancestral state (Ya)

	Estimates	Std.error
UNKNOWN	6.0220382	1.2377241
NO	0.9751567	0.3641890
YES	1.3916402	0.3719837

MODEL FIT

	Value
Support	-124.34093
AIC	258.68187
AICc	259.75330
SIC	269.31754
r squared	19.02160
SST	76.67101
SSE	62.08696

```
> model.fit(ancestor, indata2$time, seq(0.001,0.1, 0.01), seq(3.3, 3.7, 0.01), response= indata2$TLR8, me.response= NULL, fixed.fact=Group_50, fixed.cov= NULL, me.fixed.cov= NULL, mecov.fixed.cov=NULL, random.cov= NULL, me.random.cov=NULL, mecov.random.cov=NULL, intercept="root", ultrametric=TRUE, support=NULL, convergence=NULL)
```

BEST ESTIMATES & MODEL FIT – TLR8 group 75

MODEL PARAMETERS

	Estimate
Rate of adaptation	16.9060288
Phylogenetic half-life	0.0410000
Phylogenetic correction factor	0.9408495
Stationary variance	3.3600000

PRIMARY OPTIMA

UNKNOWN mapped to the root of the tree and includes the coefficent for the ancestral state (Ya)

	Estimates	Std.error
UNKNOWN	6.038627	1.2476796
NO	1.141288	0.2862024
YES	1.380672	0.6383280

MODEL FIT

	Value
Support	-124.60759
AIC	259.21519
AICc	260.28662
SIC	269.85086
r squared	18.32199
SST	75.98645
SSE	62.06422

```
> model.fit(ancestor, indata2$time, seq(0.001,0.1, 0.01), seq(3.3, 3.7, 0.01), response= indata2$TLR8,
me.response= NULL, fixed.fact=Group_75, fixed.cov= NULL, me.fixed.cov= NULL,
mecov.fixed.cov=NULL, random.cov= NULL, me.random.cov=NULL, mecov.random.cov=NULL,
intercept="root", ultrametric=TRUE, support=NULL, convergence=NULL)
```

BEST ESTIMATES & MODEL FIT – TLR8 group minus 25

MODEL PARAMETERS

	Estimate
Rate of adaptation	16.9060288
Phylogenetic half-life	0.0410000
Phylogenetic correction factor	0.9408495
Stationary variance	3.3500000

PRIMARY OPTIMA

UNKNOWN mapped to the root of the tree and includes the coefficent for the ancestral state (Ya)

	Estimates	Std.error
UNKNOWN	6.0252608	1.2429907
NO	1.2893210	0.3234544
YES	0.9619736	0.4510656

MODEL FIT

	Value
Support	-124.49214
AIC	258.98427
AICc	260.05570
SIC	269.61995
r squared	18.62558
SST	76.21327
SSE	62.01811

> model.fit(ancestor, indata2$time, seq(0.001,0.1, 0.01), seq(3.1, 3.7, 0.01), response= indata2$TLR8, me.response= NULL, fixed.fact=Group_minus_25, fixed.cov= NULL, me.fixed.cov= NULL, mecov.fixed.cov=NULL, random.cov= NULL, me.random.cov=NULL, mecov.random.cov=NULL, intercept="root", ultrametric=TRUE, support=NULL, convergence=NULL)

BEST ESTIMATES & MODEL FIT – TLR8 group minus 50

MODEL PARAMETERS

	Estimate
Rate of adaptation	13.5911212
Phylogenetic half-life	0.0510000
Phylogenetic correction factor	0.9264226
Stationary variance	3.4000000

PRIMARY OPTIMA

UNKNOWN mapped to the root of the tree and includes the coefficent for the ancestral state (Ya)

	Estimates	Std.error
UNKNOWN	5.591644	1.2010945
NO	1.074254	0.2920025
YES	1.199023	0.7783270

MODEL FIT

	Value
Support	-124.62085
AIC	259.24170
AICc	260.31312
SIC	269.87737
r squared	16.34706
SST	74.20444
SSE	62.07419

> model.fit(ancestor, indata2$time, seq(0.001,0.1, 0.01), seq(3.1, 3.7, 0.01), response= indata2$TLR8, me.response= NULL, fixed.fact=Group_minus_50, fixed.cov= NULL, me.fixed.cov= NULL, mecov.fixed.cov=NULL, random.cov= NULL, me.random.cov=NULL, mecov.random.cov=NULL, intercept="root", ultrametric=TRUE, support=NULL, convergence=NULL)

BEST ESTIMATES & MODEL FIT – TLR8 MHCII

MODEL PARAMETERS

	Estimate
Rate of adaptation	16.9060288
Phylogenetic half-life	0.0410000
Phylogenetic correction factor	0.9408495
Stationary variance	3.3200000

PRIMARY OPTIMA

UNKNOWN mapped to the root of the tree and includes the coefficent for the ancestral state (Ya)

	Estimates	Std.error
UNKNOWN	5.684552	1.2976515
No	1.644295	0.5386467
Yes	1.034836	0.3008714

MODEL FIT

	Value
Support	-124.18068
AIC	258.36135
AICc	259.43278
SIC	268.99703
r squared	19.43909
SST	76.90195
SSE	61.95291

```
> model.fit(ancestor, indata3$time, seq(0.001,0.1, 0.01), seq(3.1, 3.7, 0.01),       response= indata3$TLR8, me.response= NULL, fixed.fact=indata3$MHCII, fixed.cov= NULL, me.fixed.cov= NULL, mecov.fixed.cov=NULL, random.cov= NULL, me.random.cov=NULL, mecov.random.cov=NULL, intercept="root", ultrametric=TRUE, support=NULL, convergence=NULL)
```

BEST ESTIMATES & MODEL FIT – TLR8 group 0 + MHCII

MODEL PARAMETERS

	Estimate
Rate of adaptation	22.3595865
Phylogenetic half-life	0.0310000
Phylogenetic correction factor	0.9552765
Stationary variance	3.1000000

PRIMARY OPTIMA

UNKNOWN mapped to the root of the tree and includes the coefficent for the ancestral state (Ya)

	Estimates	Std.error
UNKNOWN	5.5081865	1.3379683
NoNO	2.7748094	0.6246435
NoYES	0.7863605	0.7210838
YesNO	1.1396624	0.4709201
YesYES	0.9948441	0.3675523

MODEL FIT

	Value
Support	-122.46304
AIC	258.92607
AICc	261.00015
SIC	273.81601
r squared	27.11397
SST	85.15327
SSE	62.06484

```
> model.fit(ancestor, indata3$time, seq(0.001,0.1, 0.01), seq(3, 3.6, 0.01),              response= indata3$TLR8, me.response= NULL, fixed.fact=Group_0_MHCII , fixed.cov= NULL, me.fixed.cov= NULL, mecov.fixed.cov=NULL, random.cov= NULL, me.random.cov=NULL, mecov.random.cov=NULL, intercept="root", ultrametric=TRUE, support=NULL, convergence=NULL)
```

BEST ESTIMATES & MODEL FIT – TLR8 group 25 + MHCII

MODEL PARAMETERS

	Estimate
Rate of adaptation	16.9060288
Phylogenetic half-life	0.0410000
Phylogenetic correction factor	0.9408495
Stationary variance	3.2800000

PRIMARY OPTIMA

UNKNOWN mapped to the root of the tree and includes the coefficent for the ancestral state (Ya)

	Estimates	Std.error
UNKNOWN	5.6499638	1.2905413
NoNO	2.0307791	0.7137641
NoYES	1.2263572	0.7395576
YesNO	0.9711936	0.6443604
YesYES	1.0524881	0.3375849

MODEL FIT

	Value
Support	-123.84106
AIC	261.68211
AICc	263.75618
SIC	276.57205
r squared	20.31684
SST	77.83978
SSE	62.02519

```
> model.fit(ancestor, indata3$time, seq(0.001,0.1, 0.01), seq(3, 3.6, 0.01),                response= indata3$TLR8, me.response= NULL, fixed.fact=Group_25_MHCII , fixed.cov= NULL, me.fixed.cov= NULL, mecov.fixed.cov=NULL, random.cov= NULL, me.random.cov=NULL, mecov.random.cov=NULL, intercept="root", ultrametric=TRUE, support=NULL, convergence=NULL)
```

BEST ESTIMATES & MODEL FIT – TLR8 group 50 + MHCII

MODEL PARAMETERS

	Estimate
Rate of adaptation	16.9060288
Phylogenetic half-life	0.0410000
Phylogenetic correction factor	0.9408495
Stationary variance	3.2100000

PRIMARY OPTIMA

UNKNOWN mapped to the root of the tree and includes the coefficent for the ancestral state (Ya)

	Estimates	Std.error
UNKNOWN	5.8463339	1.2807175
NoNO	0.4190979	0.9921280
NoYES	2.0202578	0.5889784
YesNO	1.0768274	0.3831597
YesYES	0.9711968	0.4654550

MODEL FIT

	Value
Support	-123.11637
AIC	260.23274
AICc	262.30681
SIC	275.12268
r squared	22.15806
SST	79.53722
SSE	61.91331

```
> model.fit(ancestor, indata3$time, seq(0.001,0.1, 0.01), seq(3, 3.6, 0.01),                response= indata3$TLR8, me.response= NULL, fixed.fact=Group_50_MHCII , fixed.cov= NULL, me.fixed.cov= NULL, mecov.fixed.cov=NULL, random.cov= NULL, me.random.cov=NULL, mecov.random.cov=NULL, intercept="root", ultrametric=TRUE, support=NULL, convergence=NULL)
```

BEST ESTIMATES & MODEL FIT – TLR8 group 75 + MHCII

MODEL PARAMETERS

	Estimate
Rate of adaptation	16.9060288
Phylogenetic half-life	0.0410000
Phylogenetic correction factor	0.9408495
Stationary variance	3.3100000

PRIMARY OPTIMA

UNKNOWN mapped to the root of the tree and includes the coefficent for the ancestral state (Ya)

	Estimates	Std.error
UNKNOWN	5.6715824	1.2967512
NoNO	1.5639808	0.6273580
NoYES	1.8182333	0.8824009
YesNO	1.0416566	0.3180857
YesYES	0.9790289	0.9137936

MODEL FIT

	Value
Support	-124.14774
AIC	262.29548
AICc	264.36955
SIC	277.18542
r squared	19.52468
SST	77.13428
SSE	62.07406

```
> model.fit(ancestor, indata3$time, seq(0.001,0.1, 0.01), seq(3.3, 4.3, 0.01),                response= indata3$TLR8, me.response= NULL, fixed.fact=Group_75_MHCII , fixed.cov= NULL, me.fixed.cov= NULL, mecov.fixed.cov=NULL, random.cov= NULL, me.random.cov=NULL, mecov.random.cov=NULL, intercept="root", ultrametric=TRUE, support=NULL, convergence=NULL)
```

BEST ESTIMATES & MODEL FIT - TLR8 group minus 50 + MHCII

MODEL PARAMETERS

	Estimate
Rate of adaptation	16.9060288
Phylogenetic half-life	0.0410000
Phylogenetic correction factor	0.9408495
Stationary variance	3.3200000

PRIMARY OPTIMA

UNKNOWN mapped to the root of the tree and includes the coefficent for the ancestral state (Ya)

	Estimates	Std.error
UNKNOWN	5.7152705	1.3068208
NoNO	1.5995507	0.5845279
NoYES	1.8856347	1.3390323
YesNO	1.0424670	0.3184890
YesYES	0.9703738	0.9168541

MODEL FIT

	Value
Support	-124.15854
AIC	262.31708
AICc	264.39115
SIC	277.20702
r squared	19.49666
SST	76.90195
SSE	61.90863

```
> model.fit(ancestor, indata3$time, seq(0.001,0.1, 0.01), seq(3, 3.6, 0.01),                response= indata3$TLR8, me.response= NULL, fixed.fact=Group_minus_50_MHCII , fixed.cov= NULL, me.fixed.cov= NULL, mecov.fixed.cov=NULL, random.cov= NULL, me.random.cov=NULL, mecov.random.cov=NULL,  intercept="root", ultrametric=TRUE, support=NULL, convergence=NULL)
```

BEST ESTIMATES & MODEL FIT – TLR8 group minus 25 + MHCII

MODEL PARAMETERS

	Estimate
Rate of adaptation	16.9060288
Phylogenetic half-life	0.0410000
Phylogenetic correction factor	0.9408495
Stationary variance	3.2600000

PRIMARY OPTIMA

UNKNOWN mapped to the root of the tree and includes the coefficent for the ancestral state (Ya)

	Estimates	Std.error
UNKNOWN	5.4474577	1.3064438
NoNO	2.0547246	0.6663428
NoYES	0.9743304	0.8417862
YesNO	1.0640415	0.3624955
YesYES	0.9768794	0.5240148

MODEL FIT

	Value
Support	-123.64645
AIC	261.29290
AICc	263.36698
SIC	276.18284
r squared	20.81549
SST	78.31732
SSE	62.01519

```
> model.fit(ancestor, indata3$time, seq(0.001,0.1, 0.01), seq(3, 3.6, 0.01),                response= indata3$TLR8, me.response= NULL, fixed.fact=Group_minus_25_MHCII , fixed.cov= NULL, me.fixed.cov= NULL, mecov.fixed.cov=NULL, random.cov= NULL, me.random.cov=NULL, mecov.random.cov=NULL,  intercept="root", ultrametric=TRUE, support=NULL, convergence=NULL)
```

BEST ESTIMATES & MODEL FIT – TLR9 phylogeny

MODEL PARAMETERS

	Estimate
Rate of adaptation	2.7725887
Phylogenetic half-life	0.2500000
Phylogenetic correction factor	0.6618683
Stationary variance	3.5000000

PRIMARY OPTIMA

	Estimate	Std.error
Theta_global	1.410625	0.4140082

MODEL FIT

	Value
Support	-1.187489e+02
AIC	2.434977e+02
AICc	2.439115e+02
SIC	2.498791e+02
r squared	-2.289925e-14
SST	6.205816e+01
SSE	6.205816e+01

```
> model.fit(ancestor, indata3$time, seq(0.00,0.6, 0.01), seq(1, 10, 0.1), response= indata3$TLR9, me.response= NULL, fixed.fact=NULL, fixed.cov= NULL, me.fixed.cov= NULL, mecov.fixed.cov=NULL, random.cov= NULL, me.random.cov=NULL, mecov.random.cov=NULL,  intercept="root", ultrametric=TRUE, support=NULL, convergence=NULL)
```

BEST ESTIMATES & MODEL FIT – TLR9 group 0

MODEL PARAMETERS

	Estimate
Rate of adaptation	4.3052620
Phylogenetic half-life	0.1610000
Phylogenetic correction factor	0.7708612
Stationary variance	2.6900000

PRIMARY OPTIMA

UNKNOWN mapped to the root of the tree and includes the coefficent for the ancestral state (Ya)

	Estimates	Std.error
UNKNOWN	4.90548398	0.9785545
NO	1.03000046	0.5615206
YES	-0.02680546	0.4932277

MODEL FIT

	Value
Support	-113.58672
AIC	237.17343
AICc	238.24486
SIC	247.80910
r squared	19.98517
SST	77.39867
SSE	61.93042

```
> model.fit(ancestor, indata2$time, seq(0.001,0.24, 0.01), seq(2.4, 3, 0.01), response= indata2$TLR9, me.response= NULL, fixed.fact=Group_0, fixed.cov= NULL, me.fixed.cov= NULL, mecov.fixed.cov=NULL, random.cov= NULL, me.random.cov=NULL, mecov.random.cov=NULL, intercept="root", ultrametric=TRUE, support=NULL, convergence=NULL)
```

BEST ESTIMATES & MODEL FIT – TLR9 group 25

MODEL PARAMETERS

	Estimate
Rate of adaptation	4.3052620
Phylogenetic half-life	0.1610000
Phylogenetic correction factor	0.7708612
Stationary variance	2.7800000

PRIMARY OPTIMA

UNKNOWN mapped to the root of the tree and includes the coefficent for the ancestral state (Ya)

	Estimates	Std.error
UNKNOWN	4.7905910	1.0008819
NO	0.8909195	0.7503926
YES	0.3067360	0.4442190

MODEL FIT

	Value
Support	-114.63289
AIC	239.26577
AICc	240.33720
SIC	249.90144
r squared	17.23875
SST	74.89296
SSE	61.98235

```
> model.fit(ancestor, indata2$time, seq(0.001,0.24, 0.01), seq(2.4, 3, 0.01), response= indata2$TLR9, me.response= NULL, fixed.fact=Group_25, fixed.cov= NULL, me.fixed.cov= NULL, mecov.fixed.cov=NULL, random.cov= NULL, me.random.cov=NULL, mecov.random.cov=NULL, intercept="root", ultrametric=TRUE, support=NULL, convergence=NULL)
```

BEST ESTIMATES & MODEL FIT – TLR9 group 50

MODEL PARAMETERS

	Estimate
Rate of adaptation	4.0534923
Phylogenetic half-life	0.1710000
Phylogenetic correction factor	0.7575823
Stationary variance	2.8200000

PRIMARY OPTIMA

UNKNOWN mapped to the root of the tree and includes the coefficent for the ancestral state (Ya)

	Estimates	Std.error
UNKNOWN	4.7104356	1.0032812
NO	0.2136656	0.5339074
YES	0.5316584	0.5847253

MODEL FIT

	Value
Support	-114.80147
AIC	239.60293
AICc	240.67436
SIC	250.23860
r squared	15.48955
SST	73.48147
SSE	62.09952

```
> model.fit(ancestor, indata2$time, seq(0.001,0.2, 0.01), seq(2.4, 3, 0.01), response= indata2$TLR9, me.response= NULL, fixed.fact=Group_50, fixed.cov= NULL, me.fixed.cov= NULL, mecov.fixed.cov=NULL, random.cov= NULL, me.random.cov=NULL, mecov.random.cov=NULL, intercept="root", ultrametric=TRUE, support=NULL, convergence=NULL)
```

BEST ESTIMATES & MODEL FIT – TLR9 group 75

MODEL PARAMETERS

	Estimate
Rate of adaptation	4.3052620
Phylogenetic half-life	0.1610000
Phylogenetic correction factor	0.7708612
Stationary variance	2.7200000

PRIMARY OPTIMA

UNKNOWN mapped to the root of the tree and includes the coefficent for the ancestral state (Ya)

	Estimates	Std.error
UNKNOWN	4.9086983	0.9841078
NO	0.2402895	0.4375494
YES	1.4586448	0.8695819

MODEL FIT

	Value
Support	-113.99901
AIC	237.99802
AICc	239.06945
SIC	248.63369
r squared	18.91388
SST	76.54501
SSE	62.06738

```
> model.fit(ancestor, indata2$time, seq(0.001,0.28, 0.01), seq(2, 3.4, 0.01), response= indata2$TLR9, me.response= NULL, fixed.fact=Group_75, fixed.cov= NULL, me.fixed.cov= NULL, mecov.fixed.cov=NULL, random.cov= NULL, me.random.cov=NULL, mecov.random.cov=NULL, intercept="root", ultrametric=TRUE, support=NULL, convergence=NULL)
```

BEST ESTIMATES & MODEL FIT – TLR9 group minus 25

MODEL PARAMETERS

	Estimate
Rate of adaptation	4.0534923
Phylogenetic half-life	0.1710000
Phylogenetic correction factor	0.7575823
Stationary variance	2.7800000

PRIMARY OPTIMA

UNKNOWN mapped to the root of the tree and includes the coefficent for the ancestral state (Ya)

	Estimates	Std.error
UNKNOWN	4.6624669	0.9972899
NO	0.6239572	0.5167347
YES	-0.1019681	0.6185419

MODEL FIT

	Value
Support	-114.35663
AIC	238.71326
AICc	239.78469
SIC	249.34893
r squared	16.69357
SST	74.53875
SSE	62.09557

```
> model.fit(ancestor, indata2$time, seq(0.001,0.24, 0.01), seq(2.4, 3, 0.01), response= indata2$TLR9, me.response= NULL, fixed.fact=Group_minus_25, fixed.cov= NULL, me.fixed.cov= NULL, mecov.fixed.cov=NULL, random.cov= NULL, me.random.cov=NULL, mecov.random.cov=NULL, intercept="root", ultrametric=TRUE, support=NULL, convergence=NULL)
```

BEST ESTIMATES & MODEL FIT – TLR9 group minus 50

MODEL PARAMETERS

	Estimate
Rate of adaptation	4.0534923
Phylogenetic half-life	0.1710000
Phylogenetic correction factor	0.7575823
Stationary variance	2.8000000

PRIMARY OPTIMA

UNKNOWN mapped to the root of the tree and includes the coefficent for the ancestral state (Ya)

	Estimates	Std.error
UNKNOWN	4.7207924	0.9997175
NO	0.4531282	0.4657740
YES	-0.4061054	0.9834336

MODEL FIT

	Value
Support	-114.54596
AIC	239.09192
AICc	240.16335
SIC	249.72760
r squared	16.18313
SST	74.00633
SSE	62.02979

```
> model.fit(ancestor, indata2$time, seq(0.001,0.24, 0.01), seq(2.4, 3, 0.01), response= indata2$TLR9, me.response= NULL, fixed.fact=Group_minus_50, fixed.cov= NULL, me.fixed.cov= NULL, mecov.fixed.cov=NULL, random.cov= NULL, me.random.cov=NULL, mecov.random.cov=NULL, intercept="root", ultrametric=TRUE, support=NULL, convergence=NULL)
```

BEST ESTIMATES & MODEL FIT – TLR9 + MHC

MODEL PARAMETERS

Estimate

Rate of adaptation 4.9159375

Phylogenetic half-life 0.1410000

Phylogenetic correction factor 0.7980708

Stationary variance 2.6600000

PRIMARY OPTIMA

UNKNOWN mapped to the root of the tree and includes the coefficent for the ancestral state (Ya)

Estimates Std.error

UNKNOWN 4.8358547 1.0086785

No 2.1206591 1.0813497

Yes 0.4504188 0.3750874

MODEL FIT

Value

Support -113.96842

AIC 237.93685

AICc 239.00827

SIC 248.57252

r squared 22.41454

SST 80.00114

SSE 62.06925

> model.fit(ancestor, indata3$time, seq(0.001,0.24, 0.01), seq(2.5, 3, 0.01), response= indata3$TLR9, me.response= NULL, fixed.fact=indata3$MHCII, fixed.cov= NULL, me.fixed.cov= NULL, mecov.fixed.cov=NULL, random.cov= NULL, me.random.cov=NULL, mecov.random.cov=NULL, intercept="root", ultrametric=TRUE, support=NULL, convergence=NULL)

BEST ESTIMATES & MODEL FIT – TLR9 group 0 + MHCII

MODEL PARAMETERS

	Estimate
Rate of adaptation	8.5573726
Phylogenetic half-life	0.0810000
Phylogenetic correction factor	0.8831642
Stationary variance	1.9400000

PRIMARY OPTIMA

UNKNOWN mapped to the root of the tree and includes the coefficent for the ancestral state (Ya)

	Estimates	Std.error
UNKNOWN	5.2355351	0.9269600
NoNO	5.3595659	0.7952621
NoYES	0.6606602	0.7439025
YesNO	0.9759444	0.3906908
YesYES	0.8283597	0.3103422

MODEL FIT

	Value
Support	-106.20238
AIC	226.40476
AICc	228.47883
SIC	241.29470
r squared	53.52633
SST	133.68217
SSE	62.12702

```
> model.fit(ancestor, indata3$time,  seq(0.001,0.24, 0.01), seq(1.5, 2.5, 0.01),                response= indata3$TLR9, me.response= NULL, fixed.fact=Group_0_MHCII , fixed.cov= NULL, me.fixed.cov= NULL, mecov.fixed.cov=NULL, random.cov= NULL, me.random.cov=NULL, mecov.random.cov=NULL, intercept="root", ultrametric=TRUE, support=NULL, convergence=NULL)
```

BEST ESTIMATES & MODEL FIT – TLR9 group 25 + MHCII

MODEL PARAMETERS

	Estimate
Rate of adaptation	5.7284891
Phylogenetic half-life	0.1210000
Phylogenetic correction factor	0.8260016
Stationary variance	2.4100000

PRIMARY OPTIMA

UNKNOWN mapped to the root of the tree and includes the coefficent for the ancestral state (Ya)

	Estimates	Std.error
UNKNOWN	5.2169514	0.9779169
NoNO	3.7279511	1.0997363
NoYES	0.8922586	1.0921411
YesNO	0.2904620	0.6776396
YesYES	0.6353561	0.3476031

MODEL FIT

	Value
Support	-111.52928
AIC	237.05856
AICc	239.13263
SIC	251.94850
r squared	32.69036
SST	92.10708
SSE	61.99694

> model.fit(ancestor, indata3$time, seq(0.001,0.24, 0.01), seq(2.2, 3, 0.01), response= indata3$TLR9, me.response= NULL, fixed.fact=Group_25_MHCII , fixed.cov= NULL, me.fixed.cov= NULL, mecov.fixed.cov=NULL, random.cov= NULL, me.random.cov=NULL, mecov.random.cov=NULL, intercept="root", ultrametric=TRUE, support=NULL, convergence=NULL)

BEST ESTIMATES & MODEL FIT – TLR9 group 50 + MHCII

MODEL PARAMETERS

	Estimate
Rate of adaptation	5.2911999
Phylogenetic half-life	0.1310000
Phylogenetic correction factor	0.8119587
Stationary variance	2.6000000

PRIMARY OPTIMA

UNKNOWN mapped to the root of the tree and includes the coefficent for the ancestral state (Ya)

	Estimates	Std.error
UNKNOWN	5.0964524	1.0108911
NoNO	1.0000830	1.5794960
NoYES	2.5091732	1.0470249
YesNO	0.5173308	0.4309232
YesYES	0.4629452	0.5190083

MODEL FIT

	Value
Support	-113.51586
AIC	241.03171
AICc	243.10579
SIC	255.92165
r squared	25.71577
SST	83.35805
SSE	61.92189

```
> model.fit(ancestor, indata3$time,  seq(0.001,0.24, 0.01), seq(2.2, 3, 0.01),                response= indata3$TLR9, me.response= NULL, fixed.fact=Group_50_MHCII , fixed.cov= NULL, me.fixed.cov= NULL, mecov.fixed.cov=NULL, random.cov= NULL, me.random.cov=NULL, mecov.random.cov=NULL, intercept="root", ultrametric=TRUE, support=NULL, convergence=NULL)
```

BEST ESTIMATES & MODEL FIT – TLR9 group 75 + MHCII

MODEL PARAMETERS

	Estimate
Rate of adaptation	5.7284891
Phylogenetic half-life	0.1210000
Phylogenetic correction factor	0.8260016
Stationary variance	2.4600000

PRIMARY OPTIMA

UNKNOWN mapped to the root of the tree and includes the coefficent for the ancestral state (Ya)

	Estimates	Std.error
UNKNOWN	5.1795508	0.9855519
NoNO	1.5265383	0.9921389
NoYES	4.2145472	1.3239198
YesNO	0.5368118	0.3411561
YesYES	0.8591806	0.8849256

MODEL FIT

	Value
Support	-112.15606
AIC	238.31212
AICc	240.38620
SIC	253.20206
r squared	31.31560
SST	90.23499
SSE	61.97736

```
> model.fit(ancestor, indata3$time,  seq(0.001,0.24, 0.01), seq(2.2, 3, 0.01),                response= indata3$TLR9, me.response= NULL, fixed.fact=Group_75_MHCII , fixed.cov= NULL, me.fixed.cov= NULL, mecov.fixed.cov=NULL, random.cov= NULL, me.random.cov=NULL, mecov.random.cov=NULL, intercept="root", ultrametric=TRUE, support=NULL, convergence=NULL)
```

BEST ESTIMATES & MODEL FIT – TLR9 group minus 25 + MHCII

MODEL PARAMETERS

	Estimate
Rate of adaptation	8.5573726
Phylogenetic half-life	0.0810000
Phylogenetic correction factor	0.8831642
Stationary variance	1.9900000

PRIMARY OPTIMA

UNKNOWN mapped to the root of the tree and includes the coefficent for the ancestral state (Ya)

	Estimates	Std.error
UNKNOWN	5.2313863	0.9393885
NoNO	4.9401744	0.7576044
NoYES	0.3912585	0.8023749
YesNO	0.8823702	0.3001230
YesYES	0.8908535	0.4302339

MODEL FIT

	Value
Support	-106.99338
AIC	227.98675
AICc	230.06083
SIC	242.87669
r squared	52.32525
SST	130.32332
SSE	62.13131

> model.fit(ancestor, indata3$time, seq(0.001,0.24, 0.01), seq(1.5, 3.6, 0.01), response= indata3$TLR9, me.response= NULL, fixed.fact=Group_minus_25_MHCII , fixed.cov= NULL, me.fixed.cov= NULL, mecov.fixed.cov=NULL, random.cov= NULL, me.random.cov=NULL, mecov.random.cov=NULL, intercept="root", ultrametric=TRUE, support=NULL, convergence=NULL)

BEST ESTIMATES & MODEL FIT – TLR9 group minus 50 + MHCII

MODEL PARAMETERS

	Estimate
Rate of adaptation	5.2911999
Phylogenetic half-life	0.1310000
Phylogenetic correction factor	0.8119587
Stationary variance	2.4800000

PRIMARY OPTIMA

UNKNOWN mapped to the root of the tree and includes the coefficent for the ancestral state (Ya)

	Estimates	Std.error
UNKNOWN	4.8799470	0.9823439
NoNO	2.9307760	1.0429763
NoYES	-0.6770935	1.7392722
YesNO	0.5417445	0.3585765
YesYES	0.4117317	0.9292239

MODEL FIT

	Value
Support	-112.04018
AIC	238.08036
AICc	240.15443
SIC	252.97030
r squared	29.16907
SST	87.39151
SSE	61.90021

```
> model.fit(ancestor, indata3$time,  seq(0.001,0.24, 0.01), seq(2.2, 2.7, 0.01),                  response= indata3$TLR9, me.response= NULL, fixed.fact=Group_minus_50_MHCII , fixed.cov= NULL, me.fixed.cov= NULL, mecov.fixed.cov=NULL, random.cov= NULL, me.random.cov=NULL, mecov.random.cov=NULL,  intercept="root", ultrametric=TRUE, support=NULL, convergence=NULL)
```

BEST ESTIMATES & MODEL FIT – TLR22 phylogeny

MODEL PARAMETERS

	Estimate
Rate of adaptation	8.6643398
Phylogenetic half-life	0.0800000
Phylogenetic correction factor	0.8846043
Stationary variance	59.0000000

PRIMARY OPTIMA

	Estimate	Std.error
Theta_global	3.740316	1.070306

MODEL FIT

	Value
Support	-211.93005
AIC	429.86009
AICc	430.27389
SIC	436.24150
r squared	0.00000
SST	61.79337
SSE	61.79337

```
> model.fit(ancestor, indata3$time, seq(0.00,0.5, 0.005), seq(30, 80, 1), response= indata3$TLR22, me.response= NULL, fixed.fact=NULL, fixed.cov= NULL, me.fixed.cov= NULL, mecov.fixed.cov=NULL, random.cov= NULL, me.random.cov=NULL, mecov.random.cov=NULL,  intercept="root", ultrametric=TRUE, support=NULL, convergence=NULL)
```

BEST ESTIMATES & MODEL FIT – TLR22 group 0

MODEL PARAMETERS

	Estimate
Rate of adaptation	17.3286795
Phylogenetic half-life	0.0400000
Phylogenetic correction factor	0.9422922
Stationary variance	49.4800000

PRIMARY OPTIMA

UNKNOWN mapped to the root of the tree and includes the coefficent for the ancestral state (Ya)

	Estimates	Std.error
UNKNOWN	15.276753	4.837481
NO	5.317817	1.555059
YES	1.698537	1.320510

MODEL FIT

	Value
Support	-207.98755
AIC	425.97510
AICc	427.04653
SIC	436.61077
r squared	13.99005
SST	72.07980
SSE	61.99579

```
> model.fit(ancestor, indata2$time, seq(0.01,0.07, 0.01), seq(49.3, 49.6, 0.01),  response= indata2$TLR22, me.response= NULL, fixed.fact=Group_0, fixed.cov= NULL, me.fixed.cov= NULL, mecov.fixed.cov=NULL, random.cov= NULL, me.random.cov=NULL, mecov.random.cov=NULL, intercept="root", ultrametric=TRUE, support=NULL, convergence=NULL)
```

BEST ESTIMATES & MODEL FIT – TLR22 group 25

MODEL PARAMETERS

	Estimate
Rate of adaptation	13.8629436
Phylogenetic half-life	0.0500000
Phylogenetic correction factor	0.9278653
Stationary variance	52.3700000

PRIMARY OPTIMA

UNKNOWN mapped to the root of the tree and includes the coefficent for the ancestral state (Ya)

	Estimates	Std.error
UNKNOWN	15.274336	4.757888
NO	3.363055	1.982522
YES	2.832286	1.248532

MODEL FIT

	Value
Support	-209.393675
AIC	428.787350
AICc	429.858779
SIC	439.423022
r squared	8.857167
SST	68.031163
SSE	62.005530

```
> model.fit(ancestor, indata2$time, seq(0.01,0.07, 0.01), seq(49.3, 53, 0.01),  response= indata2$TLR22, me.response= NULL, fixed.fact=Group_25, fixed.cov= NULL, me.fixed.cov= NULL, mecov.fixed.cov=NULL, random.cov= NULL, me.random.cov=NULL, mecov.random.cov=NULL, intercept="root", ultrametric=TRUE, support=NULL, convergence=NULL)
```

BEST ESTIMATES & MODEL FIT – TLR22 group 50

MODEL PARAMETERS

	Estimate
Rate of adaptation	17.3286795
Phylogenetic half-life	0.0400000
Phylogenetic correction factor	0.9422922
Stationary variance	49.6500000

PRIMARY OPTIMA

UNKNOWN mapped to the root of the tree and includes the coefficent for the ancestral state (Ya)

	Estimates	Std.error
UNKNOWN	16.059778	4.801144
NO	1.538135	1.404394
YES	4.949255	1.431323

MODEL FIT

	Value
Support	-208.09311
AIC	426.18622
AICc	427.25765
SIC	436.82190
r squared	13.69667
SST	71.83300
SSE	61.99427

```
> model.fit(ancestor, indata2$time, seq(0.01,0.07, 0.01), seq(49.3, 49.9, 0.01),  response= indata2$TLR22, me.response= NULL, fixed.fact=Group_50, fixed.cov= NULL, me.fixed.cov= NULL, mecov.fixed.cov=NULL, random.cov= NULL, me.random.cov=NULL, mecov.random.cov=NULL, intercept="root", ultrametric=TRUE, support=NULL, convergence=NULL)
```

BEST ESTIMATES & MODEL FIT – TLR22 group 75

MODEL PARAMETERS

	Estimate
Rate of adaptation	17.3286795
Phylogenetic half-life	0.0400000
Phylogenetic correction factor	0.9422922
Stationary variance	43.3500000

PRIMARY OPTIMA

UNKNOWN mapped to the root of the tree and includes the coefficent for the ancestral state (Ya)

	Estimates	Std.error
UNKNOWN	14.896336	4.502874
NO	1.840849	1.025818
YES	10.526348	2.283833

MODEL FIT

	Value
Support	-203.89235
AIC	417.78470
AICc	418.85613
SIC	428.42038
r squared	24.63373
SST	82.27239
SSE	62.00563

```
> model.fit(ancestor, indata2$time, seq(0.01,0.07, 0.01), seq(43.1, 43.6, 0.01),  response= indata2$TLR22, me.response= NULL, fixed.fact=Group_75, fixed.cov= NULL, me.fixed.cov= NULL, mecov.fixed.cov=NULL, random.cov= NULL, me.random.cov=NULL, mecov.random.cov=NULL, intercept="root", ultrametric=TRUE, support=NULL, convergence=NULL)
```

BEST ESTIMATES & MODEL FIT – TLR22 group minus 25

MODEL PARAMETERS

	Estimate
Rate of adaptation	13.8629436
Phylogenetic half-life	0.0500000
Phylogenetic correction factor	0.9278653
Stationary variance	51.9400000

PRIMARY OPTIMA

UNKNOWN mapped to the root of the tree and includes the coefficent for the ancestral state (Ya)

	Estimates	Std.error
UNKNOWN	15.152706	4.718837
NO	3.549613	1.308157
YES	1.880127	1.802368

MODEL FIT

	Value
Support	-209.134644
AIC	428.269288
AICc	429.340716
SIC	438.904960
r squared	9.615569
SST	68.594379
SSE	61.998639

```
> model.fit(ancestor, indata2$time, seq(0.01,0.07, 0.01), seq(51.8, 52, 0.01),  response= indata2$TLR22, me.response= NULL, fixed.fact=Group_minus_25, fixed.cov= NULL, me.fixed.cov= NULL, mecov.fixed.cov=NULL, random.cov= NULL, me.random.cov=NULL, mecov.random.cov=NULL, intercept="root", ultrametric=TRUE, support=NULL, convergence=NULL)
```

BEST ESTIMATES & MODEL FIT – TLR22 group minus 50

MODEL PARAMETERS

	Estimate
Rate of adaptation	13.8629436
Phylogenetic half-life	0.0500000
Phylogenetic correction factor	0.9278653
Stationary variance	52.1700000

PRIMARY OPTIMA

UNKNOWN mapped to the root of the tree and includes the coefficent for the ancestral state (Ya)

	Estimates	Std.error
UNKNOWN	15.305218	4.721236
NO	3.189032	1.140366
YES	1.442420	3.043186

MODEL FIT

	Value
Support	-209.275032
AIC	428.550065
AICc	429.621493
SIC	439.185736
r squared	9.205321
SST	68.291969
SSE	62.005474

```
> model.fit(ancestor, indata2$time, seq(0.01,0.07, 0.01), seq(52, 52.3, 0.01),  response= indata2$TLR22, me.response= NULL, fixed.fact=Group_minus_50, fixed.cov= NULL, me.fixed.cov= NULL, mecov.fixed.cov=NULL, random.cov= NULL, me.random.cov=NULL, mecov.random.cov=NULL, intercept="root", ultrametric=TRUE, support=NULL, convergence=NULL)
```

BEST ESTIMATES & MODEL FIT – TLR22 + MHCII

MODEL PARAMETERS

	Estimate
Rate of adaptation	23.1049060
Phylogenetic half-life	0.0300000
Phylogenetic correction factor	0.9567191
Stationary variance	49.6700000

PRIMARY OPTIMA

UNKNOWN mapped to the root of the tree and includes the coefficent for the ancestral state (Ya)

	Estimates	Std.error
UNKNOWN	15.449406	5.296066
No	6.339786	1.905175
Yes	2.395662	1.159599

MODEL FIT

	Value
Support	-208.46144
AIC	426.92288
AICc	427.99431
SIC	437.55855
r squared	14.52989
SST	72.54634
SSE	62.00544

```
> model.fit(ancestor, indata3$time, seq(0.01,0.07, 0.01), seq(46.8, 50, 0.01),       response= indata3$TLR22, me.response= NULL, fixed.fact=indata3$MHCII, fixed.cov= NULL, me.fixed.cov= NULL, mecov.fixed.cov=NULL, random.cov= NULL, me.random.cov=NULL, mecov.random.cov=NULL, intercept="root", ultrametric=TRUE, support=NULL, convergence=NULL)
```

BEST ESTIMATES & MODEL FIT – TLR22 group 0 + MHCII

MODEL PARAMETERS

	Estimate
Rate of adaptation	23.1049060
Phylogenetic half-life	0.0300000
Phylogenetic correction factor	0.9567191
Stationary variance	47.4500000

PRIMARY OPTIMA

UNKNOWN mapped to the root of the tree and includes the coefficent for the ancestral state (Ya)

	Estimates	Std.error
UNKNOWN	14.370374	5.268514
NoNO	8.044236	2.418499
NoYES	4.010130	2.812764
YesNO	4.281291	1.842147
YesYES	1.248559	1.437704

MODEL FIT

	Value
Support	-207.04178
AIC	428.08356
AICc	430.15763
SIC	442.97350
r squared	18.35575
SST	75.94050
SSE	62.00105

```
> model.fit(ancestor, indata3$time, seq(0.01,0.05, 0.01), seq(44, 50, 0.01),                    response= indata3$TLR22, me.response= NULL, fixed.fact=Group_0_MHCII , fixed.cov= NULL, me.fixed.cov= NULL, mecov.fixed.cov=NULL, random.cov= NULL, me.random.cov=NULL, mecov.random.cov=NULL, intercept="root", ultrametric=TRUE, support=NULL, convergence=NULL)
```

BEST ESTIMATES & MODEL FIT – TLR22 group 25 + MHCII

MODEL PARAMETERS

	Estimate
Rate of adaptation	23.1049060
Phylogenetic half-life	0.0300000
Phylogenetic correction factor	0.9567191
Stationary variance	48.6300000

PRIMARY OPTIMA

UNKNOWN mapped to the root of the tree and includes the coefficent for the ancestral state (Ya)

	Estimates	Std.error
UNKNOWN	15.1219338	5.251783
NoNO	7.9505573	2.532362
NoYES	4.5746238	2.642823
YesNO	0.9828537	2.468415
YesYES	2.7853999	1.295901

MODEL FIT

	Value
Support	-207.80551
AIC	429.61103
AICc	431.68510
SIC	444.50097
r squared	16.31934
SST	74.09781
SSE	62.00554

> model.fit(ancestor, indata3$time, seq(0.01,0.05, 0.01), seq(44, 50, 0.01), response= indata3$TLR22, me.response= NULL, fixed.fact=Group_25_MHCII , fixed.cov= NULL, me.fixed.cov= NULL, mecov.fixed.cov=NULL, random.cov= NULL, me.random.cov=NULL, mecov.random.cov=NULL, intercept="root", ultrametric=TRUE, support=NULL, convergence=NULL)

BEST ESTIMATES & MODEL FIT – TLR22 group 50 + MHCII

MODEL PARAMETERS

	Estimate
Rate of adaptation	23.1049060
Phylogenetic half-life	0.0300000
Phylogenetic correction factor	0.9567191
Stationary variance	48.1500000

PRIMARY OPTIMA

UNKNOWN mapped to the root of the tree and includes the coefficent for the ancestral state (Ya)

	Estimates	Std.error
UNKNOWN	15.633445	5.238683
NoNO	5.158900	3.555368
NoYES	6.712135	2.101357
YesNO	1.129272	1.480123
YesYES	4.255788	1.794038

MODEL FIT

	Value
Support	-207.49638
AIC	428.99275
AICc	431.06683
SIC	443.88269
r squared	17.14967
SST	74.83648
SSE	62.00228

> model.fit(ancestor, indata3$time, seq(0.01,0.06, 0.01), seq(44, 60, 0.01), response= indata3$TLR22, me.response= NULL, fixed.fact=Group_50_MHCII , fixed.cov= NULL, me.fixed.cov= NULL, mecov.fixed.cov=NULL, random.cov= NULL, me.random.cov=NULL, mecov.random.cov=NULL, intercept="root", ultrametric=TRUE, support=NULL, convergence=NULL)

BEST ESTIMATES & MODEL FIT – TLR22 group 75 + MHCII

MODEL PARAMETERS

	Estimate
Rate of adaptation	23.1049060
Phylogenetic half-life	0.0300000
Phylogenetic correction factor	0.9567191
Stationary variance	40.5000000

PRIMARY OPTIMA

UNKNOWN mapped to the root of the tree and includes the coefficent for the ancestral state (Ya)

	Estimates	Std.error
UNKNOWN	15.032341	4.794099
NoNO	5.026109	2.023606
NoYES	9.151258	2.856095
YesNO	1.109897	1.108735
YesYES	13.008162	3.184843

MODEL FIT

	Value
Support	-202.16960
AIC	418.33921
AICc	420.41328
SIC	433.22915
r squared	30.23004
SST	88.97227
SSE	62.07592

```
> model.fit(ancestor, indata3$time, seq(0.01,0.06, 0.01), seq(38, 40.5, 0.01), response= indata3$TLR22, me.response= NULL, fixed.fact=Group_75_MHCII , fixed.cov= NULL, me.fixed.cov= NULL, mecov.fixed.cov=NULL, random.cov= NULL, me.random.cov=NULL, mecov.random.cov=NULL, intercept="root", ultrametric=TRUE, support=NULL, convergence=NULL)
```

BEST ESTIMATES & MODEL FIT – TLR22 group minus 25 + MHCII

MODEL PARAMETERS

	Estimate
Rate of adaptation	23.1049060
Phylogenetic half-life	0.0300000
Phylogenetic correction factor	0.9567191
Stationary variance	48.9400000

PRIMARY OPTIMA

UNKNOWN mapped to the root of the tree and includes the coefficent for the ancestral state (Ya)

	Estimates	Std.error
UNKNOWN	14.917277	5.330581
NoNO	7.146068	2.309934
NoYES	4.798604	3.160920
YesNO	2.993395	1.400046
YesYES	1.150329	2.021938

MODEL FIT

	Value
Support	-207.99899
AIC	429.99798
AICc	432.07206
SIC	444.88793
r squared	15.79543
SST	73.62846
SSE	61.99852

```
> model.fit(ancestor, indata3$time, seq(0.01,0.05, 0.01), seq(44, 50, 0.01),                    response= indata3$TLR22, me.response= NULL, fixed.fact=Group_minus_25_MHCII , fixed.cov= NULL, me.fixed.cov= NULL, mecov.fixed.cov=NULL, random.cov= NULL, me.random.cov=NULL, mecov.random.cov=NULL,  intercept="root", ultrametric=TRUE, support=NULL, convergence=NULL)
```

BEST ESTIMATES & MODEL FIT – TLR22 group minus 50 + MHCII

MODEL PARAMETERS

	Estimate
Rate of adaptation	23.1049060
Phylogenetic half-life	0.0300000
Phylogenetic correction factor	0.9567191
Stationary variance	49.0300000

PRIMARY OPTIMA

UNKNOWN mapped to the root of the tree and includes the coefficent for the ancestral state (Ya)

	Estimates	Std.error
UNKNOWN	14.916259	5.298721
NoNO	6.992132	2.040317
NoYES	2.361683	5.013570
YesNO	2.506920	1.219826
YesYES	1.480402	3.506411

MODEL FIT

	Value
Support	-208.05874
AIC	430.11749
AICc	432.19156
SIC	445.00743
r squared	15.63298
SST	73.49330
SSE	62.00411

```
> model.fit(ancestor, indata3$time, seq(0.01,0.05, 0.01), seq(44, 50, 0.01),                    response= indata3$TLR22, me.response= NULL, fixed.fact=Group_minus_50_MHCII , fixed.cov= NULL, me.fixed.cov= NULL, mecov.fixed.cov=NULL, random.cov= NULL, me.random.cov=NULL, mecov.random.cov=NULL,  intercept="root", ultrametric=TRUE, support=NULL, convergence=NULL)
```

BEST ESTIMATES & MODEL FIT – TLR25 phylogeny

MODEL PARAMETERS

	Estimate
Rate of adaptation	11.7482573
Phylogenetic half-life	0.0590000
Phylogenetic correction factor	0.9148817
Stationary variance	2.7000000

PRIMARY OPTIMA

	Estimate	Std.error
Theta_global	1.021704	0.2229397

MODEL FIT

	Value
Support	-117.4721
AIC	240.9441
AICc	241.3579
SIC	247.3255
r squared	0.0000
SST	62.6399
SSE	62.6399

```
> model.fit(ancestor, indata3$time, seq(0.00,0.15, 0.001), seq(1, 10, 0.1), response= indata3$TLR25, me.response= NULL, fixed.fact=NULL, fixed.cov= NULL, me.fixed.cov= NULL, mecov.fixed.cov=NULL, random.cov= NULL, me.random.cov=NULL, mecov.random.cov=NULL,  intercept="root", ultrametric=TRUE, support=NULL, convergence=NULL)
```

BEST ESTIMATES & MODEL FIT – TLR25 group 0

MODEL PARAMETERS

	Estimate
Rate of adaptation	17.3286795
Phylogenetic half-life	0.0400000
Phylogenetic correction factor	0.9422922
Stationary variance	2.1500000

PRIMARY OPTIMA

UNKNOWN mapped to the root of the tree and includes the coefficent for the ancestral state (Ya)

	Estimates	Std.error
UNKNOWN	4.8602440	1.0083787
NO	1.0701899	0.3241538
YES	0.5886049	0.2752620

MODEL FIT

	Value
Support	-110.74512
AIC	231.49023
AICc	232.56166
SIC	242.12590
r squared	21.83949
SST	79.25891
SSE	61.94917

```
> model.fit(ancestor, indata2$time, seq(0.01,0.08, 0.01), seq(1.8, 3, 0.01),   response= indata2$TLR25, me.response= NULL, fixed.fact=Group_0, fixed.cov= NULL, me.fixed.cov= NULL, mecov.fixed.cov=NULL, random.cov= NULL, me.random.cov=NULL, mecov.random.cov=NULL, intercept="root", ultrametric=TRUE, support=NULL, convergence=NULL)
```

BEST ESTIMATES & MODEL FIT – TLR25 group 25

MODEL PARAMETERS

	Estimate
Rate of adaptation	17.3286795
Phylogenetic half-life	0.0400000
Phylogenetic correction factor	0.9422922
Stationary variance	2.1700000

PRIMARY OPTIMA

UNKNOWN mapped to the root of the tree and includes the coefficent for the ancestral state (Ya)

	Estimates	Std.error
UNKNOWN	4.9454882	1.0085298
NO	1.0286538	0.3876642
YES	0.6932093	0.2495884

MODEL FIT

	Value
Support	-111.12050
AIC	232.24099
AICc	233.31242
SIC	242.87666
r squared	20.88742
SST	78.52841
SSE	62.12585

```
> model.fit(ancestor, indata2$time, seq(0.01,0.08, 0.01), seq(1.8, 3, 0.01),   response= indata2$TLR25, me.response= NULL, fixed.fact=Group_25, fixed.cov= NULL, me.fixed.cov= NULL, mecov.fixed.cov=NULL, random.cov= NULL, me.random.cov=NULL, mecov.random.cov=NULL, intercept="root", ultrametric=TRUE, support=NULL, convergence=NULL)
```

BEST ESTIMATES & MODEL FIT – TLR25 group 50

MODEL PARAMETERS

	Estimate
Rate of adaptation	17.3286795
Phylogenetic half-life	0.0400000
Phylogenetic correction factor	0.9422922
Stationary variance	2.1400000

PRIMARY OPTIMA

UNKNOWN mapped to the root of the tree and includes the coefficent for the ancestral state (Ya)

	Estimates	Std.error
UNKNOWN	4.9596281	0.9967631
NO	0.5510381	0.2915656
YES	1.0380182	0.2971563

MODEL FIT

	Value
Support	-110.69630
AIC	231.39259
AICc	232.46402
SIC	242.02827
r squared	21.96265
SST	79.62928
SSE	62.14057

```
> model.fit(ancestor, indata2$time, seq(0.01,0.08, 0.01), seq(1.8, 3, 0.01),   response= indata2$TLR25,
me.response= NULL, fixed.fact=Group_50, fixed.cov= NULL, me.fixed.cov= NULL,
mecov.fixed.cov=NULL, random.cov= NULL, me.random.cov=NULL, mecov.random.cov=NULL,
intercept="root", ultrametric=TRUE, support=NULL, convergence=NULL)
```

BEST ESTIMATES & MODEL FIT – TLR25 group 75

MODEL PARAMETERS

	Estimate
Rate of adaptation	23.1049060
Phylogenetic half-life	0.0300000
Phylogenetic correction factor	0.9567191
Stationary variance	1.9300000

PRIMARY OPTIMA

UNKNOWN mapped to the root of the tree and includes the coefficent for the ancestral state (Ya)

	Estimates	Std.error
UNKNOWN	5.2370077	1.0103873
NO	0.6168722	0.2122440
YES	2.1608119	0.4634191

MODEL FIT

	Value
Support	-107.77088
AIC	225.54177
AICc	226.61319
SIC	236.17744
r squared	32.25999
SST	91.51601
SSE	61.99296

```
> model.fit(ancestor, indata2$time, seq(0.01,0.08, 0.01), seq(1.8, 2, 0.01),   response= indata2$TLR25,
me.response= NULL, fixed.fact=Group_75, fixed.cov= NULL, me.fixed.cov= NULL,
mecov.fixed.cov=NULL, random.cov= NULL, me.random.cov=NULL, mecov.random.cov=NULL,
intercept="root", ultrametric=TRUE, support=NULL, convergence=NULL)
```

BEST ESTIMATES & MODEL FIT – TLR25 group minus 25

MODEL PARAMETERS

	Estimate
Rate of adaptation	17.3286795
Phylogenetic half-life	0.0400000
Phylogenetic correction factor	0.9422922
Stationary variance	2.1800000

PRIMARY OPTIMA

UNKNOWN mapped to the root of the tree and includes the coefficent for the ancestral state (Ya)

	Estimates	Std.error
UNKNOWN	4.9748519	1.0074679
NO	0.8926867	0.2601707
YES	0.5871671	0.3633712

MODEL FIT

	Value
Support	-111.15389
AIC	232.30778
AICc	233.37921
SIC	242.94345
r squared	20.80208
SST	78.16819
SSE	61.90758

```
> model.fit(ancestor, indata2$time, seq(0.01,0.08, 0.01), seq(1.8, 3, 0.01),   response= indata2$TLR25, me.response= NULL, fixed.fact=Group_minus_25, fixed.cov= NULL, me.fixed.cov= NULL, mecov.fixed.cov=NULL, random.cov= NULL, me.random.cov=NULL, mecov.random.cov=NULL, intercept="root", ultrametric=TRUE, support=NULL, convergence=NULL)
```

BEST ESTIMATES & MODEL FIT – TLR25 group minus 50

MODEL PARAMETERS

	Estimate
Rate of adaptation	17.3286795
Phylogenetic half-life	0.0400000
Phylogenetic correction factor	0.9422922
Stationary variance	2.1800000

PRIMARY OPTIMA

UNKNOWN mapped to the root of the tree and includes the coefficent for the ancestral state (Ya)

	Estimates	Std.error
UNKNOWN	5.0003525	1.0053592
NO	0.8387468	0.2265925
YES	0.4277453	0.6122499

MODEL FIT

	Value
Support	-111.19170
AIC	232.38339
AICc	233.45482
SIC	243.01907
r squared	20.70535
SST	78.16819
SSE	61.98319

```
> model.fit(ancestor, indata2$time, seq(0.01,0.08, 0.01), seq(1.8, 3, 0.01),   response= indata2$TLR25, me.response= NULL, fixed.fact=Group_minus_50, fixed.cov= NULL, me.fixed.cov= NULL, mecov.fixed.cov=NULL, random.cov= NULL, me.random.cov=NULL, mecov.random.cov=NULL, intercept="root", ultrametric=TRUE, support=NULL, convergence=NULL)
```

BEST ESTIMATES & MODEL FIT – TLR25 + MHCII

MODEL PARAMETERS

	Estimate
Rate of adaptation	23.1049060
Phylogenetic half-life	0.0300000
Phylogenetic correction factor	0.9567191
Stationary variance	2.0800000

PRIMARY OPTIMA

UNKNOWN mapped to the root of the tree and includes the coefficent for the ancestral state (Ya)

	Estimates	Std.error
UNKNOWN	4.990496	1.0837721
No	1.555048	0.3898697
Yes	0.618451	0.2372971

MODEL FIT

	Value
Support	-110.11597
AIC	230.23194
AICc	231.30337
SIC	240.86761
r squared	26.93680
SST	84.91630
SSE	62.04256

```
> model.fit(ancestor, indata3$time,  seq(0.01,0.08, 0.01), seq(1.8, 3, 0.01),       response= indata3$TLR25, me.response= NULL, fixed.fact=indata3$MHCII, fixed.cov= NULL, me.fixed.cov= NULL, mecov.fixed.cov=NULL, random.cov= NULL, me.random.cov=NULL, mecov.random.cov=NULL, intercept="root", ultrametric=TRUE, support=NULL, convergence=NULL)
```

BEST ESTIMATES & MODEL FIT – TLR25 group 0 + MHCII

MODEL PARAMETERS

	Estimate
Rate of adaptation	23.1049060
Phylogenetic half-life	0.0300000
Phylogenetic correction factor	0.9567191
Stationary variance	1.9900000

PRIMARY OPTIMA

UNKNOWN mapped to the root of the tree and includes the coefficent for the ancestral state (Ya)

	Estimates	Std.error
UNKNOWN	4.6643980	1.0789381
NoNO	2.0683599	0.4952840
NoYES	0.8537901	0.5760254
YesNO	0.7121886	0.3772530
YesYES	0.5617877	0.2944273

MODEL FIT

	Value
Support	-108.77914
AIC	231.55828
AICc	233.63235
SIC	246.44822
r squared	30.02067
SST	88.75673
SSE	62.11137

```
> model.fit(ancestor, indata3$time,  seq(0.01,0.08, 0.01), seq(1.3, 2.8, 0.01),
response= indata3$TLR25, me.response= NULL, fixed.fact=Group_0_MHCII , fixed.cov= NULL,
me.fixed.cov= NULL, mecov.fixed.cov=NULL, random.cov= NULL, me.random.cov=NULL,
mecov.random.cov=NULL,  intercept="root", ultrametric=TRUE, support=NULL, convergence=NULL)
```

BEST ESTIMATES & MODEL FIT – TLR25 group 25 + MHCII

MODEL PARAMETERS

	Estimate
Rate of adaptation	23.1049060
Phylogenetic half-life	0.0300000
Phylogenetic correction factor	0.9567191
Stationary variance	2.0300000

PRIMARY OPTIMA

UNKNOWN mapped to the root of the tree and includes the coefficent for the ancestral state (Ya)

	Estimates	Std.error
UNKNOWN	4.9060143	1.0730073
NoNO	1.9682961	0.5173944
NoYES	1.1023082	0.5399630
YesNO	0.4951979	0.5043291
YesYES	0.6525104	0.2647694

MODEL FIT

	Value
Support	-109.37207
AIC	232.74414
AICc	234.81822
SIC	247.63408
r squared	28.66923
SST	87.00783
SSE	62.06336

```
> model.fit(ancestor, indata3$time,  seq(0.01,0.08, 0.01), seq(1.3, 2.8, 0.01),
response= indata3$TLR25, me.response= NULL, fixed.fact=Group_25_MHCII , fixed.cov= NULL,
me.fixed.cov= NULL, mecov.fixed.cov=NULL, random.cov= NULL, me.random.cov=NULL,
mecov.random.cov=NULL,  intercept="root", ultrametric=TRUE, support=NULL, convergence=NULL)
```

BEST ESTIMATES & MODEL FIT – TLR25 group 50 + MHCII

MODEL PARAMETERS

	Estimate
Rate of adaptation	23.1049060
Phylogenetic half-life	0.0300000
Phylogenetic correction factor	0.9567191
Stationary variance	2.0600000

PRIMARY OPTIMA

UNKNOWN mapped to the root of the tree and includes the coefficent for the ancestral state (Ya)

	Estimates	Std.error
UNKNOWN	5.0484793	1.0835715
NoNO	1.2000166	0.7353938
NoYES	1.6665774	0.4346457
YesNO	0.4979530	0.3061494
YesYES	0.7953611	0.3710797

MODEL FIT

	Value
Support	-109.76484
AIC	233.52968
AICc	235.60376
SIC	248.41962
r squared	27.75972
SST	85.74073
SSE	61.93935

```
> model.fit(ancestor, indata3$time,  seq(0.01,0.08, 0.01), seq(1, 2.5, 0.01),                    response= indata3$TLR25, me.response= NULL, fixed.fact=Group_50_MHCII , fixed.cov= NULL, me.fixed.cov= NULL, mecov.fixed.cov=NULL, random.cov= NULL, me.random.cov=NULL, mecov.random.cov=NULL, intercept="root", ultrametric=TRUE, support=NULL, convergence=NULL)
```

BEST ESTIMATES & MODEL FIT – TLR25 group 75 + MHCII

MODEL PARAMETERS

	Estimate
Rate of adaptation	23.1049060
Phylogenetic half-life	0.0300000
Phylogenetic correction factor	0.9567191
Stationary variance	1.8500000

PRIMARY OPTIMA

UNKNOWN mapped to the root of the tree and includes the coefficent for the ancestral state (Ya)

	Estimates	Std.error
UNKNOWN	4.8155471	1.0246259
NoNO	1.0017827	0.4324982
NoYES	2.7388718	0.6104231
YesNO	0.5119824	0.2369661
YesYES	1.4982955	0.6806854

MODEL FIT

	Value
Support	-106.45249
AIC	226.90499
AICc	228.97906
SIC	241.79493
r squared	35.08048
SST	95.47346
SSE	61.98091

```
> model.fit(ancestor, indata3$time,  seq(0.01,0.08, 0.01), seq(1, 4, 0.01),                    response= indata3$TLR25, me.response= NULL, fixed.fact=Group_75_MHCII , fixed.cov= NULL, me.fixed.cov= NULL, mecov.fixed.cov=NULL, random.cov= NULL, me.random.cov=NULL, mecov.random.cov=NULL, intercept="root", ultrametric=TRUE, support=NULL, convergence=NULL)
```

BEST ESTIMATES & MODEL FIT – TLR25 group minus 25 + MHCII
MODEL PARAMETERS

	Estimate
Rate of adaptation	23.1049060
Phylogenetic half-life	0.0300000
Phylogenetic correction factor	0.9567191
Stationary variance	2.0300000

PRIMARY OPTIMA

UNKNOWN mapped to the root of the tree and includes the coefficent for the ancestral state (Ya)

	Estimates	Std.error
UNKNOWN	4.7821822	1.0856518
NoNO	1.8687699	0.4704524
NoYES	0.9558465	0.6437682
YesNO	0.6776808	0.2851401
YesYES	0.4954454	0.4117977

MODEL FIT

	Value
Support	-109.38494
AIC	232.76988
AICc	234.84396
SIC	247.65983
r squared	28.63965
SST	87.00783
SSE	62.08910

```
> model.fit(ancestor, indata3$time,  seq(0.01,0.08, 0.01), seq(1.3, 2.8, 0.01),
response= indata3$TLR25, me.response= NULL, fixed.fact=Group_minus_25_MHCII , fixed.cov= NULL,
me.fixed.cov= NULL, mecov.fixed.cov=NULL, random.cov= NULL, me.random.cov=NULL,
mecov.random.cov=NULL,  intercept="root", ultrametric=TRUE, support=NULL, convergence=NULL)
```

BEST ESTIMATES & MODEL FIT – TLR25 group minus 50 + MHCII

MODEL PARAMETERS

	Estimate
Rate of adaptation	23.1049060
Phylogenetic half-life	0.0300000
Phylogenetic correction factor	0.9567191
Stationary variance	2.0600000

PRIMARY OPTIMA

UNKNOWN mapped to the root of the tree and includes the coefficent for the ancestral state (Ya)

	Estimates	Std.error
UNKNOWN	4.9100324	1.0861097
NoNO	1.6539606	0.4182156
NoYES	0.9515187	1.0276607
YesNO	0.6639259	0.2500348
YesYES	0.2433077	0.7187294

MODEL FIT

	Value
Support	-109.76376
AIC	233.52751
AICc	235.60159
SIC	248.41745
r squared	27.76225
SST	85.74073
SSE	61.93718

```
> model.fit(ancestor, indata3$time,  seq(0.01,0.08, 0.01), seq(1.3, 2.8, 0.01),
response= indata3$TLR25, me.response= NULL, fixed.fact=Group_minus_50_MHCII , fixed.cov= NULL,
me.fixed.cov= NULL, mecov.fixed.cov=NULL, random.cov= NULL, me.random.cov=NULL,
mecov.random.cov=NULL,  intercept="root", ultrametric=TRUE, support=NULL, convergence=NULL)
```

BEST ESTIMATES & MODEL FIT – TLR9 phylogenetic effect

MODEL PARAMETERS

	Estimate
Rate of adaptation	5.7762265
Phylogenetic half-life	0.1200000
Phylogenetic correction factor	0.6305712
Stationary variance	6.3700000

PRIMARY OPTIMA

	Estimate	Std.error
Theta_global	2.324661	0.7098559

MODEL FIT

	Value
Support	-1.183494e+02
AIC	2.426988e+02
AICc	2.430988e+02
SIC	2.491755e+02
r squared	-2.221451e-14
SST	6.397104e+01
SSE	6.397104e+01

model.fit(ancestor, time, seq(0,0.5, 0.01), seq(5, 10, 0.01), response= TLR9, me.response= NULL, fixed.fact=NULL, fixed.cov= NULL, me.fixed.cov= NULL, mecov.fixed.cov=NULL, random.cov= NULL, me.random.cov=NULL, mecov.random.cov=NULL, intercept="root", ultrametric=TRUE, support=NULL, convergence=NULL)

BEST ESTIMATES & MODEL FIT – TLR9 maximum depth

MODEL PARAMETERS

Estimate

Rate of adaptation 4.3321699

Phylogenetic half-life 0.1600000

Phylogenetic correction factor 0.5517876

Stationary variance 6.1900000

Maxdepth

Predictor theta 7.176028e+02

Predictor variance 2.127460e+07

PRIMARY OPTIMA

Evolutionary regression

	Estimate	Std. Error
Intercept	2.55726	0.76584
Maxdepth	-0.00052	0.00020

Optimal regression

	Estimate	Std. Error
K	2.40257	0.75533
Maxdepth	-0.00093	0.00036

	Bias-corr. regression parameters
K	2.5572620431
Maxdepth	-0.0005178823

Decomposition of K assuming Ya = Xa to get the optimal regression intercept Bo

[1] 2.70278

(Use this as the intercept when plotting the regression line)

MODEL FIT

Support -114.709044

AIC 237.418088

AICc 238.096054

SIC 246.053621

r squared 9.512911

SST 72.640560

SSE 65.730328

model.fit(ancestor, time, seq(0.05,0.3, 0.01), seq(6.1, 6.3, 0.01), response= TLR9, me.response= NULL, fixed.fact=NULL, fixed.cov= NULL, me.fixed.cov= NULL, mecov.fixed.cov=NULL, random.cov= Maxdepth, me.random.cov=NULL, mecov.random.cov=NULL, intercept="root", ultrametric=TRUE, support=NULL, convergence=NULL)

BEST ESTIMATES & MODEL FIT – TLR22 phylogenetic effect

MODEL PARAMETERS

	Estimate
Rate of adaptation	17.3286795
Phylogenetic half-life	0.0400000
Phylogenetic correction factor	0.8526366
Stationary variance	70.6000000

PRIMARY OPTIMA

	Estimate	Std.error
Theta_global	4.499373	1.638639

MODEL FIT

	Value
Support	-214.89433
AIC	435.78867
AICc	436.18867
SIC	442.26532
r squared	0.00000
SST	64.02347
SSE	64.02347

model.fit(ancestor, time, seq(0,0.1, 0.01), seq(60, 100, 0.1), response= TLR22, me.response= NULL, fixed.fact=NULL, fixed.cov= NULL, me.fixed.cov= NULL, mecov.fixed.cov=NULL, random.cov= NULL, me.random.cov=NULL, mecov.random.cov=NULL, intercept="root", ultrametric=TRUE, support=NULL, convergence=NULL)

BEST ESTIMATES & MODEL FIT – TLR22 maximum depth

MODEL PARAMETERS

	Estimate
Rate of adaptation	13.8629436
Phylogenetic half-life	0.0500000
Phylogenetic correction factor	0.8200624
Stationary variance	72.1100000

	Maxdepth
Predictor theta	7.176028e+02
Predictor variance	2.127460e+07

PRIMARY OPTIMA

Evolutionary regression

	Estimate	Std. Error
Intercept	2.89181	2.06756
Maxdepth	0.00176	0.00100

Optimal regression

	Estimate	Std. Error
K	2.96982	2.04058
Maxdepth	0.00217	0.00122

	Bias-corr. regression parameters
K	2.891805411
Maxdepth	0.001762484

Decomposition of K assuming Ya = Xa to get the optimal regression intercept Bo

[1] 2.689926

(Use this as the intercept when plotting the regression line)

MODEL FIT

	Value
Support	-213.507480

AIC 435.014959

AICc 435.692926

SIC 443.650492

r squared 4.743322

SST 67.049714

SSE 63.869330

model.fit(ancestor, time, seq(0.03,0.06, 0.01), seq(72, 73, 0.01), response= TLR22, me.response= NULL, fixed.fact=NULL, fixed.cov= NULL, me.fixed.cov= NULL, mecov.fixed.cov=NULL, random.cov= Maxdepth, me.random.cov=NULL, mecov.random.cov=NULL, intercept="root", ultrametric=TRUE, support=NULL, convergence=NULL)

Supplementary table 3 Diversifying selection analyses - results from MEME (mixed effects model of evolution)

TLR3 Selection model

*	AC	1	AT
-	*	AC	1
-	-	*	AT
-	-	-	*

MEME results significance level 0.05

Codon	a	ß-	Pr[ß=ß-]	ß+	Pr[ß=ß+]	p-value	q-value
11	0.3127	0.1638	0.7918	5.9282	0.2082	0.0190	1
14	0	0	0.8081	14.4583	0.1919	0.0061	0.7157
23	0.6057	0.3156	0.9597	65.4143	0.0403	0.0393	1
28	0.2113	0	0.7628	1.8349	0.2372	0.0332	1
31	0.5751	0.0995	0.7785	5.3002	0.2215	0.0222	1
40	0.5945	0	0.7064	3.8732	0.2936	0.0403	1
75	0.6945	0.0492	0.7472	8.6920	0.2528	0.0024	0.3717
84	0	0	0.8840	4.2079	0.1160	0.0278	1
97	0.2739	0	0.7741	3.1438	0.2259	0.0461	1
107	0.3551	0.0407	0.8448	9.0952	0.1552	0.0124	0.9796
145	0.1071	0.0489	0.9038	21.9453	0.0962	0.0002	0.1091
162	0.2529	0.1645	0.6936	9.3461	0.3064	0.0270	1
166	0.3948	0	0.8522	45.9382	0.1478	0.0296	1
241	0.8265	0	0.9437	3728.7500	0.0563	0.0193	1
250	0	0	0.8077	2.3165	0.1923	0.0397	1
339	0.8311	0.3103	0.9587	610.8070	0.0413	0.0393	1
394	0	0	0.3060	2.1701	0.6940	0.0023	0.5341
413	0	0	0.7623	8.2909	0.2377	0.0083	0.7863
414	0.2407	0.0924	0.9640	3333.1100	0.0360	0.0176	1

TLR9 1 Selection model

*	AC	1	AT
-	*	AC	CT
-	-	*	AT
-	-	-	*

MEME results significance level 0.05

Codon	a	ß-	Pr[ß=ß-]	ß+	Pr[ß=ß+]	p-value	q-value
4	0	0	0.5120	5.8732	0.4880	0.0095	0.4929
8	0.1018	0	0.3788	1.6997	0.6212	0.0137	0.4764
103	0.1744	0.1744	0.8010	102.6540	0.1990	0	0.0004
127	0.9911	0.5416	0.8643	18.6733	0.1357	0.0443	1
143	0.4189	0	0.9092	10.6899	0.0908	0.0099	0.4398
154	0.1298	0.1298	0	2.0668	1	0.0308	0.8002
172	0.4630	0	0.9787	110.3240	0.0213	0.0223	0.6317
198	0	0	0.9248	29.8538	0.0752	0	0.0004
229	1.6669	0.3283	0.8809	37.5264	0.1191	0.0133	0.5172
230	0.5929	0.1874	0.8734	20.4878	0.1266	0.0011	0.1116
233	1.0481	0.2233	0.8062	12.3231	0.1938	0.0080	0.4991
239	0.7056	0	0.8957	19.4503	0.1043	0.0045	0.3518
311	0.5993	0.5667	0.9270	38.9681	0.0730	0.0168	0.5227

TLR9 2 Selection model

*	AC	1	AT
-	*	AC	1
-	-	*	AT
-	-	-	*

MEME results significance level 0.05

Codon	a	ß-	Pr[ß=ß-]	ß+	Pr[ß=ß+]	p-value	q-value
2	0	0	0.3209	4.6936	0.6791	0.0189	0.4587
17	0.6945	0.2940	0.9768	304.7330	0.0232	0.0012	0.1025
89	1.4672	0	0.9587	25.6757	0.0413	0.0219	0.4636
92	0.0364	0.0364	0.9812	81.0526	0.0188	0.0203	0.4593
96	0.6880	0	0.9607	24.9478	0.0393	0.0010	0.1087
127	0	0	0	1.0409	1	0.0018	0.0994
137	0.3491	0	0.7918	4.5849	0.2082	0.0032	0.1193
149	0.0859	0	0.7482	7.4237	0.2518	0.0263	0.5242
150	0.2284	0.2284	0.9255	21.6380	0.0745	0.0453	0.7314
173	0.7268	0	0.5647	4.3052	0.4353	0.0270	0.5078
177	0.5215	0.0340	0.8707	27.7355	0.1293	0.0144	0.3749
179	1.2577	0.2557	0.9752	189.8810	0.0248	0.0111	0.3418
181	0.2659	0.0664	0.8804	15.5179	0.1196	0.0017	0.1152
198	0	0	0.6072	9.6686	0.3928	0.0024	0.1139
206	0.5019	0	0.8494	5.0683	0.1506	0.0370	0.6279
218	0.2645	0	0.7688	5.8491	0.2312	0.0009	0.1477
242	0.1431	0	0.8267	10.8630	0.1733	0.0005	0.1734
266	0.2078	0.2078	0.8620	10.6094	0.1380	0.0078	0.2635
268	0.6004	0.1383	0.8141	20.7451	0.1859	0.0030	0.1292
270	0.4748	0.0468	0.9506	40057.0000	0.0494	0.0121	0.3411
276	0.5561	0.2925	0.8429	13.0299	0.1571	0.0312	0.5559
320	0.7292	0.7292	0.8764	17.8974	0.1236	0.0488	0.7517

TLR25 1 Selection model

*	AC	1	AC
-	*	AC	1
-	-	*	AC
-	-	-	*

MEME results significance level 0.05

Codon	a	ß-	Pr[ß=ß-]	ß+	Pr[ß=ß+]	p-value	q-value
5	0.3218	0	0.7954	3.4903	0.2046	0.0392	1
57	0.0621	0	0.5173	2.8724	0.4827	0.0489	1

TLR25 2 Selection model

*	AC	1	AT

-	*	AT	AC
-	-	*	AT
-	-	-	*

MEME results significance level 0.05

Codon	a	ß-	Pr[ß=ß-]	ß+	Pr[ß=ß+]	p-value	q-value
36	0	0	0.1088	1.7258	0.8912	0.0112	0.4987
57	0	0	0	1.4135	1	0.0377	1
67	2.1751	0.2475	0.8339	45.4946	0.1661	0.0094	0.6245
79	0	0	0	1.4386	1	0.0057	0.7617

TLR25 3 Selection model

*	AC	1	AT
-	*	AC	CT
-	-	*	AT
-	-	-	*

MEME results significance level 0.05

Codon	a	ß-	Pr[ß=ß-]	ß+	Pr[ß=ß+]	p-value	q-value
96	0.9550	0.4347	0.8928	30.1618	0.1072	0.0175	0.9273
114	0	0	0.7961	6.6317	0.2039	0.0036	0.3848
117	0	0	0.6883	9.3362	0.3117	0.0305	1
193	0	0	0.9450	29.9778	0.0550	0	0.0073
195	0.7467	0.7467	0.9034	51.5451	0.0966	0.0123	0.8694

TLR25 4 Selection model

*	AC	1	AC
-	*	AC	1
-	-	*	AC
-	-	-	*

MEME results significance level 0.05

Codon	a	ß-	Pr[ß=ß-]	ß+	Pr[ß=ß+]	p-value	q-value
1	0.2197	0	0.8691	10.1151	0.1309	0.0272	0.9193
10	0.8679	0	0.6632	17.3242	0.3368	0.0065	0.5455
16	0.5304	0.2878	0.8765	18.6946	0.1235	0.0020	0.3359
37	0.2254	0.2254	0.7115	10.9954	0.2885	0.0232	0.9822
62	0.8570	0	0.6552	5.0907	0.3448	0.0305	0.8587
71	0.3064	0	0.9683	120.9230	0.0317	0.0087	0.4929
86	1.5487	0	0.9651	29.8710	0.0349	0.0410	0.9900

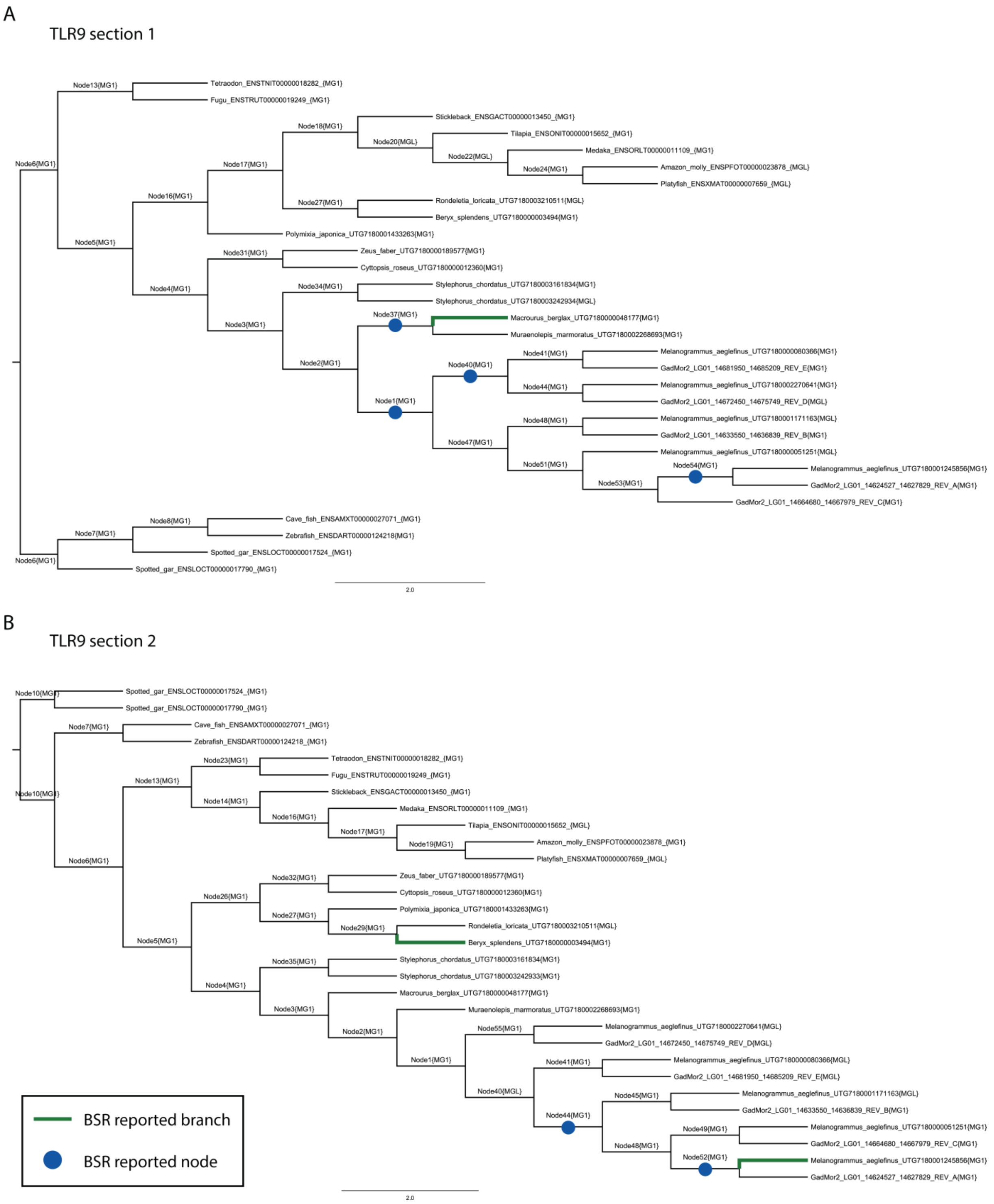

Supplementary figure 1 Tree derived from the BSR selection analysis where significantly reported nodes and branches likely to have experience diversifying selection are highlighted. There are two trees representing section 1 (A) and section 2 (B) of the overall TLR9 alignments. The trees were rooted on the sequences from Spotted gar and ordered in FigTree. Colors added in Illustrator.

TLR25 section 3

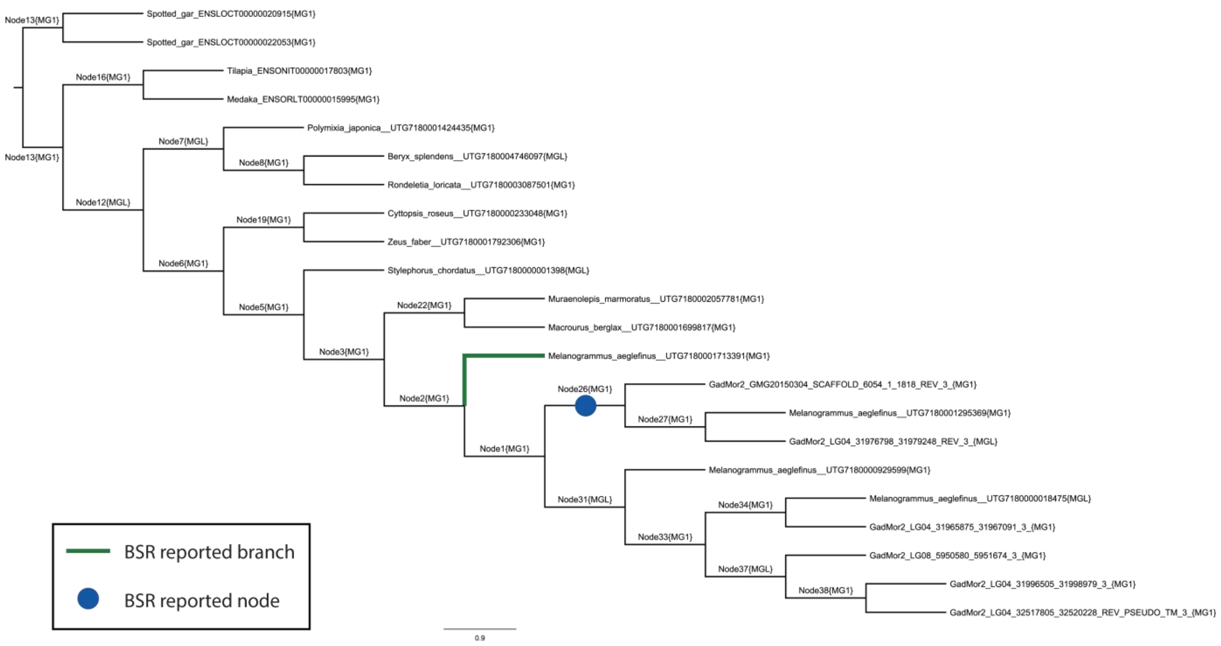

Supplementary figure 2 Tree derived from the BSR selection analysis where significantly reported nodes and branches likely to have experience diversifying selection are highlighted. The tree represents the BSR analysis performed on section 3 of the overall TLR25 alignment. The tree was rooted on the sequences from Spotted gar and ordered in FigTree. Colors added in Illustrator.
